# Recent achievements in nano-based technologies for ocular disease diagnosis and treatment, review and update

**DOI:** 10.1186/s12951-022-01567-7

**Published:** 2022-08-02

**Authors:** Mehrdad Afarid, Shirin Mahmoodi, Roghayyeh Baghban

**Affiliations:** 1 Poostchi Ophthalmology Research Center, Department of Ophthalmology, School of Medicine, Shiraz University of Medical Sciences, Shiraz, Iran; 2 Department of Medical Biotechnology, School of Medicine, Fasa University of Medical Sciences, Fasa, Iran

**Keywords:** Nanotechnology, Ocular diseases, Diagnosis, Treatment

## Abstract

Ocular drug delivery is one of the most challenging endeavors among the various available drug delivery systems. Despite having suitable drugs for the treatment of ophthalmic disease, we have not yet succeeded in achieving a proper drug delivery approach with the least adverse effects. Nanotechnology offers great opportunities to overwhelm the restrictions of common ocular delivery systems, including low therapeutic effects and adverse effects because of invasive surgery or systemic exposure. The present review is dedicated to highlighting and updating the recent achievements of nano-based technologies for ocular disease diagnosis and treatment. While further effort remains, the progress illustrated here might pave the way to new and very useful ocular nanomedicines.

## Introduction

**Fig. 1:**
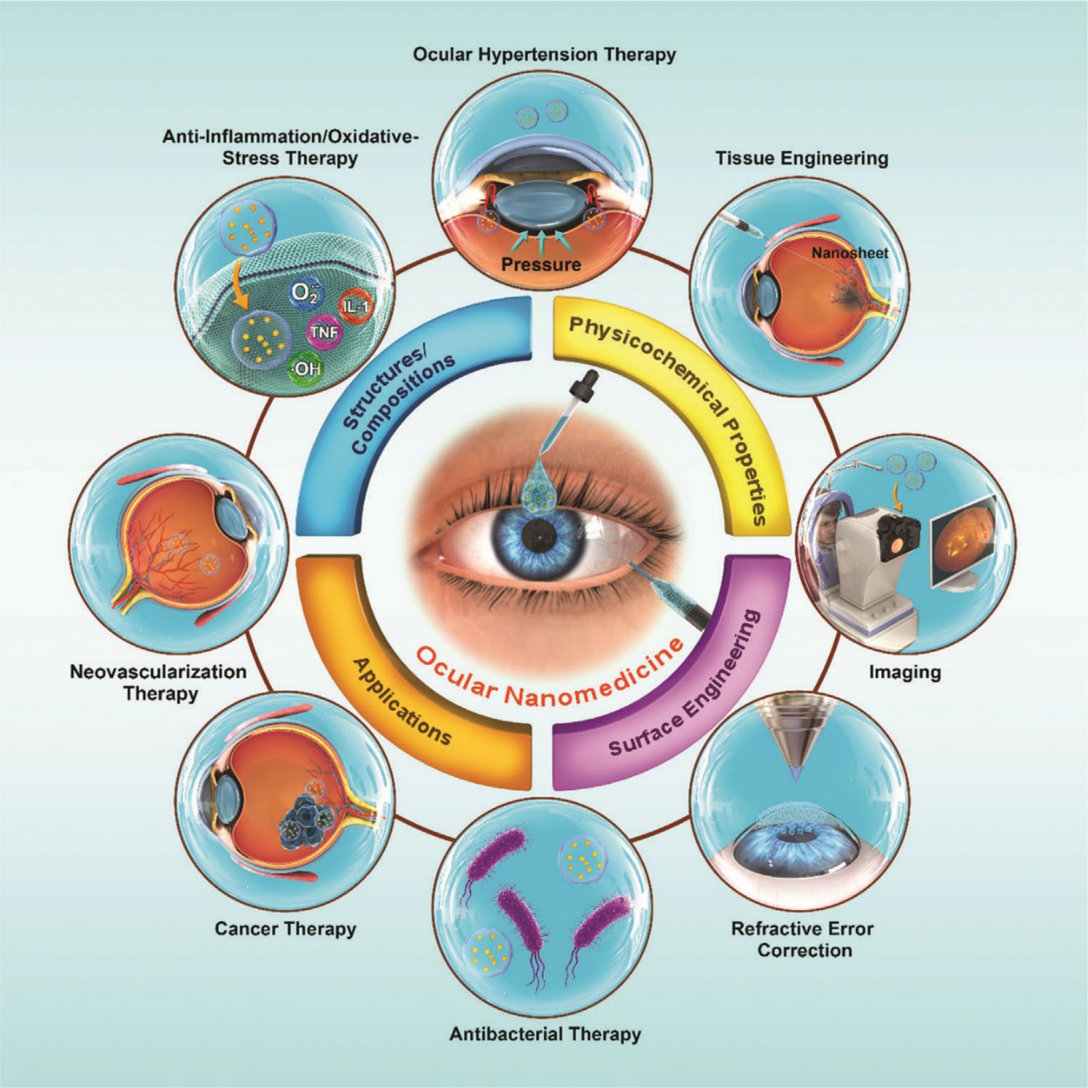
Schematic representation of ocular nanomedicine for various biomedical uses in ophthalmology [15]

Based on the investigations conducted by the World Health Organization (WHO) in 2015, almost 217 million people aged 18 years or older globally are suffering from various ocular disorders that could lead to vision impairment and finally cause permanent blindness [1, 2]. In the last ten years, immense preclinical and clinical studies have been performed for the development of therapeutics for different ophthalmic disorders, for example, diabetic retinopathy, age-related macular degeneration (AMD), glaucoma, cataracts, and uveitis [3]. Considerable accomplishments have been made in the innovation of ophthalmic pathological mechanisms and eye disease management. Nevertheless, owing to the unique anatomical and physiological features of the eye, detection and treatment of these diseases face many challenges. The common therapeutic approaches rarely could entirely return vision loss or diagnosis of severe ophthalmic disorders at an early stage [4, 5]. Thus, improved diagnostic and therapeutic emerging methods for eye diseases have received much attention. Nanotechnology, as a current hot topic and high potential technology, has an important effect on many fields associated with engineering, chemistry, medicine, and, biology. In the last ten years, this technology has attracted considerable research attention [6–10]. Nanoscience refers at the study of the biological, chemical, and physical properties of materials at the nanometer scale. Nanotechnology involves the construction and use of materials with at least one dimension in the nanometer scale [11]. Certain nanoscale materials show exceptional mechanical, electrical, optical, magnetic, and chemical characteristics. These properties can be utilized to improve the physicochemical and biological characteristics of drugs and drug delivery systems [12–14]. The application of nanotechnology in the diagnosis and treatment of ocular disorders has also made rapid progress (Fig. 1).

Nanocapsules, nanohydrogels, nanoliposomes, nanomicelles, niosomes, cubosomes and nanoparticles (NPs) are among the most common nanotechnology-based ophthalmic delivery systems offering some benefits over current diagnostics and therapeutics approaches [16–19]. Due to their special features and possible uses in biology and medicine, nanomaterials were developed to revolutionize the detection and treatment of many disorders [20–23]. Nowadays, NPs are widely utilized for the successful delivery of many drugs, peptides, vaccines, etc. [24]. Due to their acceptable therapeutic toxicity, controlled-release, and also nanometer-scale dimensions, NPs yield encouraging results even in minimal concentrations and possess lesser adverse effects compared with conventional chemotherapy drugs [5, 25]. Hence, NPs are more attractive objects for use in NP-contained contact lens implants, nanostructured devices, and films for ophthalmic drug delivery [5].

In this review, we have focused on recent achievements in nanotechnology-based systems for ocular disease diagnosis and treatment. First, the main challenges in common ocular drug delivery systems are introduced. Then, we will discuss the applications of nano-based materials for ophthalmic drug delivery. In the following, magnetic-based materials (iron oxide-based materials, gold-based materials, silica-based materials), polymeric-based materials (lipid-based materials and polysaccharide-based materials) for the treatment of ocular diseases are highlighted and reviewed. Then, nano-biomaterials for regenerative ophthalmology are discussed. Finally, we will summarize the advantages and disadvantages of nanocarriers, as well as, their safety and toxicity for ophthalmic drug delivery, and the perspective of nanotechnology in ocular diseases diagnosis and treatment.

## Challenges in the current ocular drug delivery systems

**Fig. 2:**
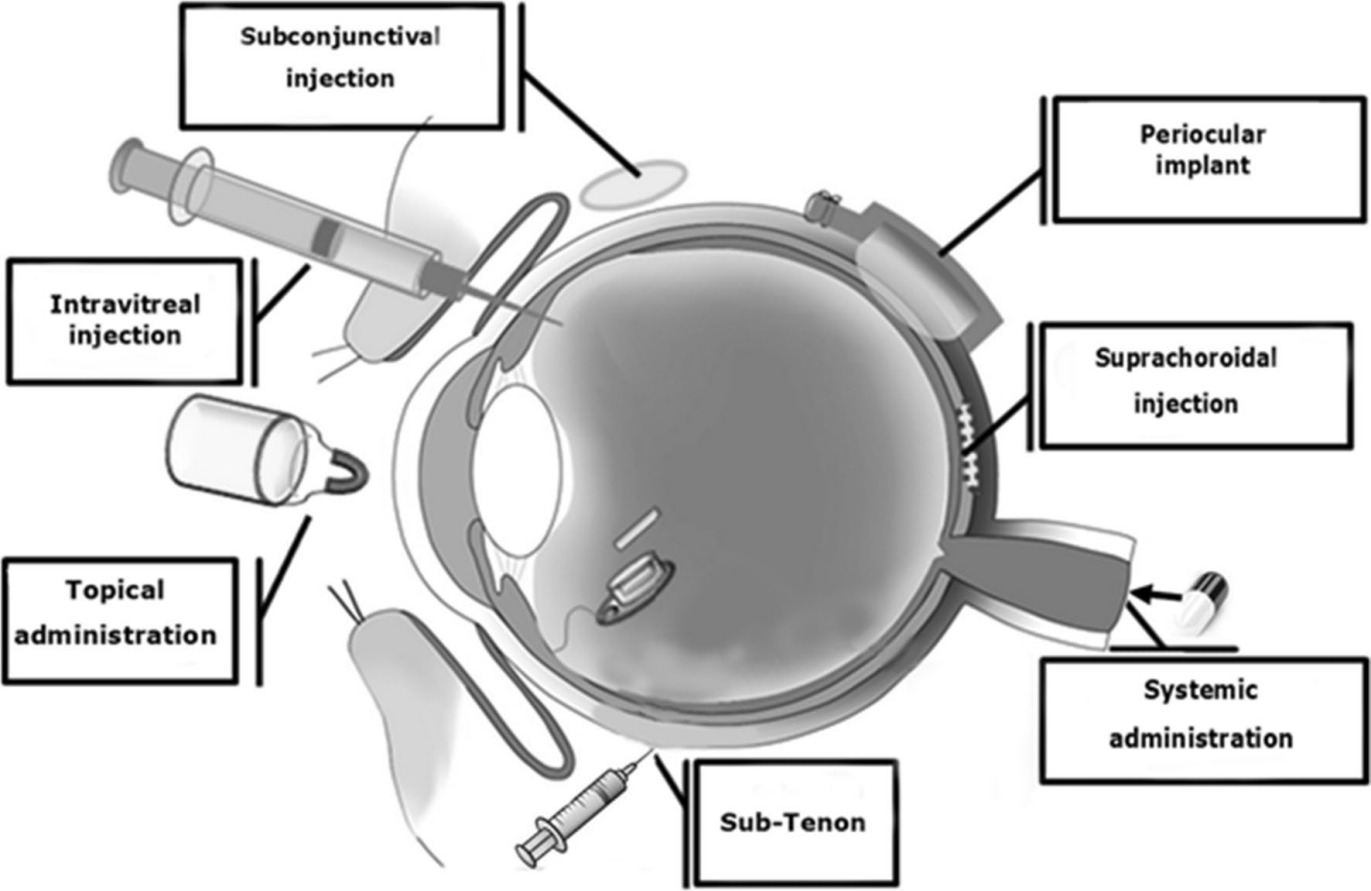
Graphical representation of the various delivery routes for ocular administration [26]

There are different administration routes for ophthalmic drug delivery that are widely used to reach the posterior segment in clinical practice (Fig. 2). These drug administration routes face many challenges. In the following, the most common methods of drug delivery to the eye and their challenges will be discussed (Table 1).

**Table 1:** Different administration routes for ocular drug delivery

Administration route	Approach	Target disease	Advantage	Challenge	Refs.
Systemic	Systemic intravenous and oral administration	Endophthalmitis, uveitis, retinitis (acute retinal necrosis), cytomegalovirus retinitis, metastasis, malignancies, episcleritis, scleritis	Very effective for the simultaneous treatment of ocular and systemic diseases. Oral administration: non-invasive, good patient compliance, no requirement to apply strict sterile conditions, the large availability of pharmaceutical forms, high stability of drug	Systemic side effects, high dosing causes toxicity, blood-aqueous barrier, blood-retinal barrier, bioavailability < 2%	[43, 257, 258]
Topical	Topical usage	Anterior uveitis, conjunctivitis, keratitis, blepharitis, scleritis, episcleritis	Non-invasive, painless, ease of administration, patient compliance, localized drug effects, less drug entry into the systemic circulation	Tear renewal and large lacrimal clearance, blinking, nasolacrimal drainage, ocular dynamic and static barriers, low bioavailability	[43, 259, 260]
Intravitreal injection	Direct injection of drugs into the vitreous	AMD, posterior uveitis, branched retinal vein occlusion, central retinal vein occlusion, diabetic macular edema, cystoid macular edema, uveitic macular edema, cytomegalovirus retinitis, endophthalmitis	Attaining rapid therapeutics in the vitreous humor, limited systemic side effects, sustained drug levels, avoiding blood-retinal barrier	Cataract, retinal detachment, endophthalmitis, uveitis, retinal hemorrhage, vitreous detachment, subconjunctival hemorrhage, inflammation, cataracts, increased intraocular pressure, retinal toxicity, eye pain caused by invasive procedure	[43, 261, 262]
Periocular implants	Located eyeball's outer part and used the trans-scleral route for drug delivery	AMD and uveitis	Safer and less invasive than intraocular implants, more patient friendly, decreased complications, localized drug delivery. Biodegradable implants: capability to be metabolized, eliminated via a physiological pathway, ease of construction, ability to break down into non-toxic substances, and prevent inflammation after usage	Requires surgery, restricted by some static barriers (sclera, choroid, and RPE) and eliminated by lymph and blood flow in the surrounding tissues, final burst stage of the device and uncontrolled release of the remaining drug	[263–270]
Subconjunctival	Subconjunctival injection	Glaucoma, uveitis, AMD, corneal ulcer	Greater concentrations of drug in the anterior chamber than topical administration, less invasive compared to intravitreal injection, reduced adverse effects, such as cataracts, endophthalmitis, and retinal damage	Diminished bioavailability due to elimination through systemic circulation, subconjunctival hemorrhages, the possibility of glob preformation	[36, 39, 42]
Suprachoroidal	Injected into the supracervical space	Diabetic macular edema, macular degeneration, non-infectious uveitis and ocular oncology	Avoiding various ophthalmic barriers (e.g., cornea, conjunctiva and sclera), suprachoroidal space can act as a possible reservoir within the eye, sustained-release formulations, providing a safer way with larger immunogenic/biologic agents	Suprachoroidal hemorrhage, choroidal detachment, retinal detachment, subretinal hemorrhage	[47, 271]
Sub-Tenon	Placing a formulation between the Tenon’s capsule and sclera	Chronic posterior uveitis, cystoid macular edema, diabetic macular edema	Increased permeation to the posterior eye segment, safer than intravitreal injections, no need to enter the eye and eliminating needle-associated risks, reducing drug passage to systemic circulation, prolonging the contact time with the sclera	Increased intraocular pressure, worsening cataracts, less efficacy in the treatment of uveitic macular edema in comparison with intravitreal injection, removal of the drug due to choroidal circulation, possibility of glob preformation, subconjunctival hemorrhage	[32, 49–51, 272, 273]

### Systemic administration

Systemic administration, majorly through the oral and intravenous routes, is a drug delivery method to the posterior eye segment thru the choroidal capillaries. But, the blood-retinal barrier (BRB) and blood-aqueous barrier (BAB) hamper the drug molecules' penetration. Hence, a significantly higher dose is required for drug efficacy, leading to enhanced drug toxicity [27, 28]. In some disorders mainly, systemic disorders (like rheumatologic disorders) or life-threatening systemic diseases (like endophthalmitis), systemic administration is recommended in a unique situation. But if a patient has an isolated ocular problem, it is better to hesitate systemic administration. Despite the high oral and intravenous bioavailability, the side effects of the systemic route cannot be ignored [29–31]. Therefore, systemic administration is not a preferred technique for the above-mentioned reasons, and this drug delivery method for administrating the drug to the posterior segment faces many challenges [32, 33].

### Topical administration

**Fig. 3:**
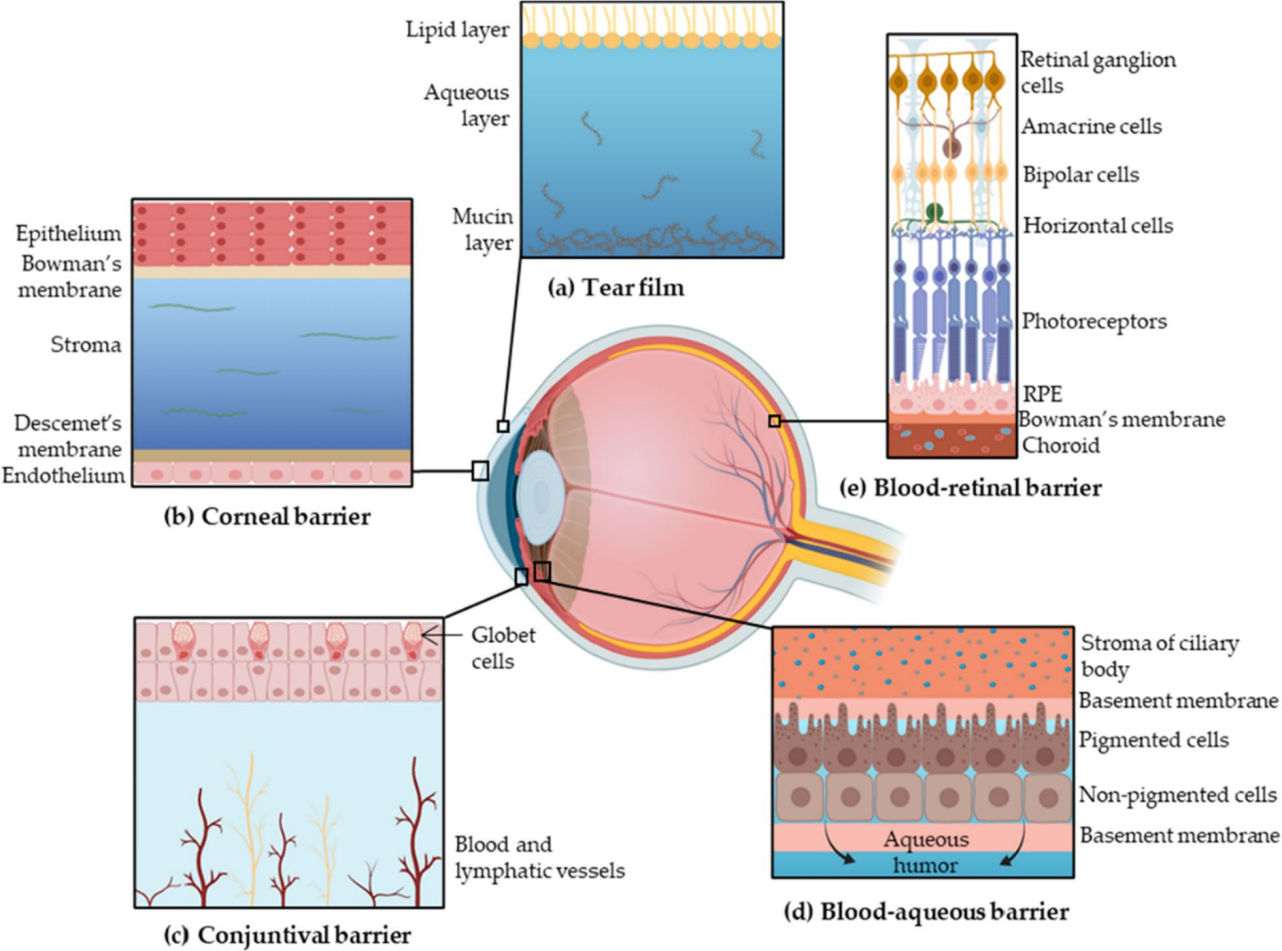
Ocular barriers to drug delivery: (**a**) the tear film is consist of the mucin, aqueous, and lipid layers; (**b**) the corneal layer is composed of the endothelium, Descemet’s membrane, stroma, Bowman’s membrane, and epithelium; (**c**) the conjunctival barrier; (**d**) the blood-aqueous barrier begins at the ciliary body stroma and is formed by the basement membrane, pigmented and non-pigmented cells and is specified with the basement membrane; (**e**) the blood-retinal barrier is composed of Bowman’s membrane, RPE, photoreceptors, horizontal cells, bipolar cells, amacrine cells, and the retinal ganglion cells [26]

One of the prevalent routes of drug administration is topical usage. In the topical route, conventional formulations e. g. ointments, eye drops, and suspensions are used for good patient compliance. Drug delivery to the targeted ocular tissues is limited by several local barriers (Fig. 3). Lacrimation and washing of drugs during lacrimation are the main challenges, and if we decided to use a drug topically, it must absorb sufficiently and rapidly before washing with tear. The other challenge is the corneal epithelium barrier. Drugs must have the capability to penetrate the corneal layers. Interaction of a drug with tear film enzymes or proteins and also with anterior chamber molecules is the other challenge. After each step, the drug must have sufficient concentration to reach the main target site [26].

### Intravitreal injection

This approach includes injecting ophthalmic drugs straight in the vitreous through pars plana with a 30G injection needle that improves drug absorption compared to topically and systemically delivered drugs. In this method, due to targeting a specific site, drug delivery to the posterior eye segment is more than systemic administration. Compared to other methods, this administration route offers greater concentrations of drugs in the retina and vitreous. Deletion of drugs after intravitreal injection is based on size [34]. While intravitreal administration presents high drug concentrations in the retina, this drug delivery method faces some technical complications, for example, intravitreal hemorrhages, endophthalmitis, trauma to the lens, and retinal detachment [35].

### Periocular implants

Periocular implants are usually placed on the surface of the eyeball, which includes the subtenon, subconjunctival, retrobulbar, posterior juxta-scleral, and peribulbar spaces. These implants utilize the trans-scleral route for drug delivery into the vitreous, retina, and choroid [36–39]. The trans-scleral route has some advantages, including a high hydration level of the sclera, a large surface area, and the high permeability of larger drugs. Furthermore, as this delivery route facilitates localized drug delivery to the needed site and is minimally invasive compared to intravitreal injection, trans-scleral delivery has appeared as a more interesting approach for the treatment of retinal disorders. Nevertheless, this approach is restricted by the several static barriers, which include the RPE, choroid, and sclera, and the flow-related elimination by blood and lymphatic vessels in the surrounding tissues [40, 41].

### Subconjunctival

The subconjunctival injection route is used for drug delivery in the anterior eye segment, reaching greater concentrations of drug in this segment compared with the topical route. This administration route has been considered a different route for the drug delivery and treatment of the retinal disorder [36, 39, 42]. It diminishes the side effects, majorly retinal damage, cataracts, and endophthalmitis [42]. The drug is administered beneath the conjunctival membrane which covers the sclera. So, the drug could avoid the conjunctiva-cornea barrier and facilitates direct access to the trans-scleral route [42], enhancing its bioavailability in aqueous humor compared to the topical route, which offers the corneal barrier as an obstacle. This administration route has many drawbacks considering the concentration that could reach the retina which can be referred to eliminate through systemic flow and the tear causing a decreased bioavailability [43].

### Suprachoroidal

The suprachoroidal administration route was presented as a potential drug delivery system to the posterior eye segment while it is not currently utilized in the clinic [41]. In this route, the drug is administrated by microneedles into the supracervical space under the inner surface of the sclera. Thereby, the pressure applied during the injection process, the formulation is dispersed all over the suprachoroidal space [44]. This approach has been examined for delivery of drugs into the posterior eye segment using surgical methods, and gave rise to a long-term treatment, where drugs can be targeted towards the retina and choroid using direct contact with the injection site [45, 46]. Disadvantages of the suprachoroidal administration route include choroidal detachment, suprachoroidal hemorrhage, difficult accessibility to the suprachoroidal space and invasive and complexity of this method [47]. Therefore, the mentioned challenges have made it difficult to use [45].

### Sub-tenon

The Subtenon route involves the administration of a drug between Tenon’s capsule and the sclera, an avascular membrane. Thus, the contact time between the injected drug and the sclera is extended [48]. The Subtenon administration route is probably safer than intravitreal injection as the method does not need to enter the eye eliminating needle-associated risks [49]. Increased intraocular pressure, worsening cataracts, and lower effectiveness in treating uveitic macular edema compared to intravitreal steroids in a large randomized controlled trial, have diminished this route’s usage with other alternatives accessible [50, 51]. Nevertheless, this delivery route remains the main tool in uveitis expert’s hands, particularly in those cases where intravitreal administration might not be possible or need exceptional attempts. As with any intraocular or periocular steroid injection, worsening cataracts and ocular hypertension are possible with subtenon or subconjunctival’s triamcinolone [52].

Although considerable research efforts have been made to improve the efficiency and overcome the limitations of common ocular drug delivery systems, many efforts still demand to be done. Thus, in order to overcome the above-mentioned adverse side effects, extensive research has been focused on the development of new therapeutic strategies for the delivery of ophthalmic drugs. Nano-based technologies have attracted great attention to overcome the challenges of current ocular drug delivery systems and promote drug delivery to the anterior and posterior parts of the eye. In the following sections, recent advances in nanotechnology-based systems for ocular disease diagnosis and treatment will be discussed.

## Applications of nano-based materials for ocular drug delivery

### The application of nanomaterials for the diagnosis of ocular diseases

In recent years, the application of nanotechnologies has been undergoing significant progress in the detection of various cancers and eye diseases. Nanoparticles, nanoliposomes, nanocapsules, nanocages, nanohydrogels, nanomicelles, and nanodendrimers are considered the most practical nanotechnology-based ophthalmic delivery systems offering numerous benefits over common diagnostics [16–19]. Due to their exceptional features and possible uses in biology and medicine, nanomaterials have been developed to revolutionize disease detection [20, 21, 23, 53]. Here, diagnostic applications of nanotechnology in ophthalmology are given.

Fundus fluorescein angiography (FFA) is the commonly applied diagnostic devise in ophthalmology for visualization of the choroid and retinal blood flow after the injection of the fluorescein sodium. Its clinical detection has been restricted owing to unusual anaphylaxis and vomiting with an incidence rate of 0.04–0.3%, and 0.6–2%, respectively [54]. The recent achievements in nanosized contrast agents have the auspicious capacity to form FFA for AMD, which may diminish the NPs adsorption in the ophthalmic tissues and improve cellular toxicity facets [55].

Due to the potential ability of nanodiscs and gold nanorods, these nanomaterials have been studied as contrast agents to improve optical coherence tomography (OCT) and increase visualization of eye structures for early detection. Gold NPs have been examined for home screening for diabetic retinopathy by a urine-based colorimetric test paper that links with a smartphone for screening biomarkers of diabetic retinopathy by capturing color with the camera. The 8-hydroxy-2’-deoxyguanosine, is an oxidative stress DNA decomposition product from hyperglycemia and is a known diabetic retinopathy/nephropathy biomarker. The gold NPs exhibited 81% specificity and 91% sensitivity. Though there was no association with the severity of diabetic retinopathy, the results showed that NPs have the capacity of screening for retinal diseases [83].

Cai et al. have produced ranibizumab-loaded double imaging and therapeutic (S-PEG-ICG-RGD-RBZ) NPs via conjugation of polyethylene glycol (PEG) with core cross-linked star polymers and also more modified by ICG and RGD for CNV. Following in vivo and in vitro evaluations, NPs did not show any cytotoxicity, genotoxicity and, apoptosis on mouse CNV (choroidal neovascularization) models and RPE (retinal pigment epithelium) cell lines, respectively. Moreover, the produced NPs prevented the VEGF-induced proliferation and tube formation. Additionally, it limited the VEGF and CD31 expression in the human cells and also repressed the CNV progression in the mouse model. Conclusively, fluorescence images of NPs in the ophthalmic fundus of the rodent eye detected the targeted fluorescence imaging of CNV regions and did not stay for a long period in the other organs [56].

**Fig. 4:**
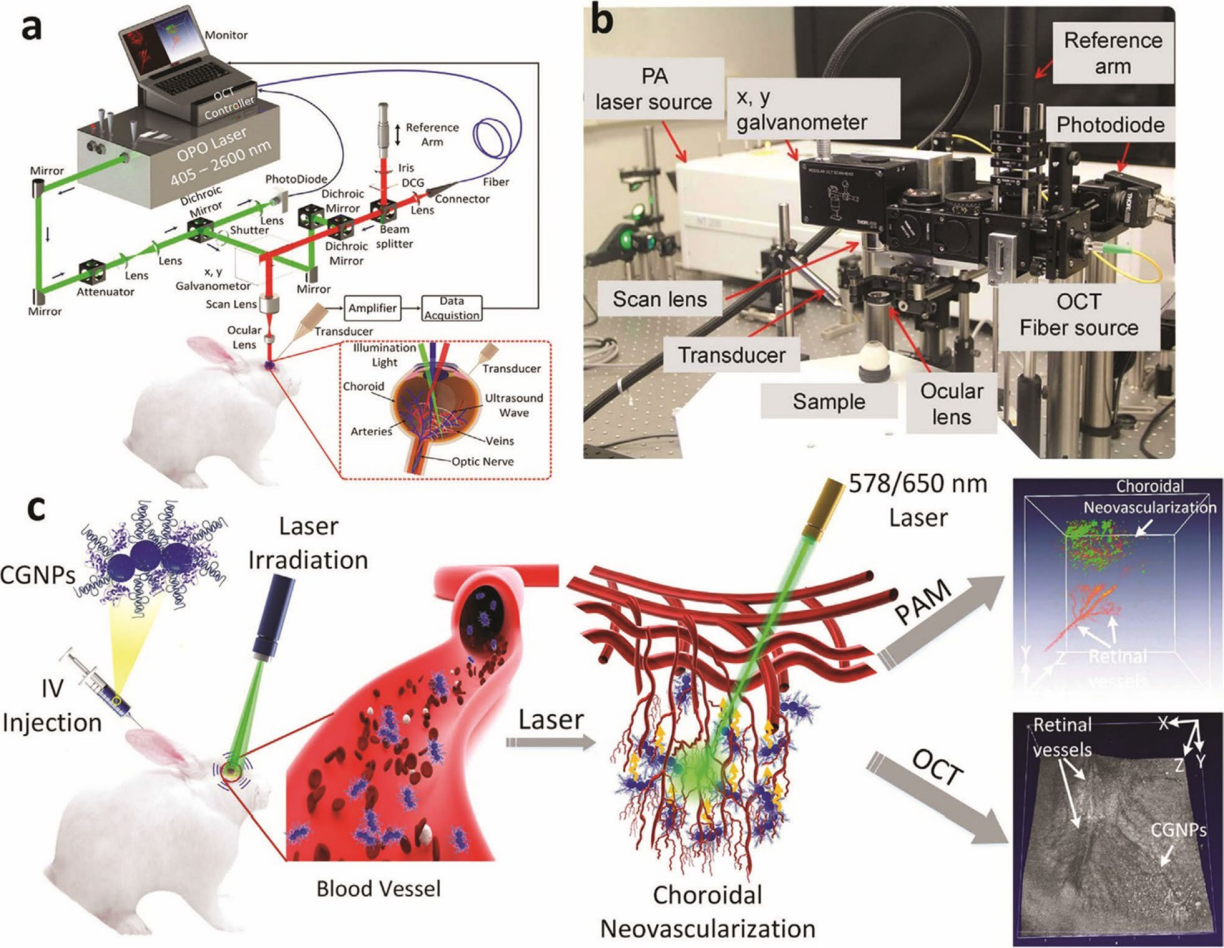
Chain-like clusters of gold nanoparticles (CGNP) increased molecular imaging for optical coherence tomography and multimodal photoacoustic microscopy (PAM). Experimental setup of OCT/PAM systems. **a** Schematic representation of the imaging technique. **b** Physical setup. In the PAM method, nanosecond excitation laser is concentrated on the retina. For multidimensional imaging, the excitation laser beam employed to induce photoacoustic signal was coaxially aligned with OCT multispectral luminescence with a center wavelength (805 and 905 nm). Using a needle-shaped hydrophone ultrasonic transducer, the produced acoustic signal was detected and the recorded data was utilized to reproduce PAM images. It employed a spectrometer for detecting the reflected OCT light that interfered with the interference intensity spectra and the reference light. By using a galvanometer, the retina was scanned. **c** Demonstration of in vivo multidimensional imaging following intravenous administration of CGNP clusters-RGD into the rabbit model. By using nanosecond-pulsed laser light at 578/650 nm, photoacoustic signals from the rabbit's retina were produced [57]

Colloidal gold nanoparticles (GNPs) are hopeful contrast agents for molecular imaging. Nguyen et al. synthesized chain-like GNPs (CGNP) clusters-RGD for increased molecular imaging via combining RGD peptides and ultra-pure CGNP clusters, showing a red-shift peak 650 nm wavelength. The designed NPs presented outstanding biocompatibility and photostability and can disassemble to help eliminate from the body. Notably, intravenous administration of CGNP clusters RGD through the marginal ear vein attached to CNV, causing a 176% increase in OCT signal and an enhancement of up to 17-fold in the photoacoustic microscopy signal, which is useful for visualization of newly formed blood vessels in the subretinal space [57] (Fig. 4).

The early diagnosis of retinoblastoma (RB) is the main feature of successful therapy [58]. Nano-based technologies propose different nanomaterials for hopeful early diagnosis and ongoing surveillance of treating cancer patients [16, 17, 53, 59]. In recent times, numerous nanosystems have been designed to increase the image quality of conventional imaging systems [60–63]. But, adequate research efforts have not been done to increase the performance of conventional ophthalmic imaging methods, including OCT, MRI, and ultrasound imaging via nanosystems [64]. However, these nanosystems have exhibited remarkable capacity for increasing the quality of imaging and detection of retinal disorders.

Moradi et al. investigated the efficacy of the brachytherapy by ultrasonic hyperthermia method in the presence of gold NPs on eye RB tumors in a rabbit model. The area of the tumor was evaluated at day zero and after 21 days by an ultrasound B-mode imaging method. There was a big difference between the tumor region changes in the combined group and the other study groups. The finding showed that gold NPs have a large capacity in various imaging systems, such as ultrasounds [65].

Vasculature visualization is one of the significant applications of AuNPs in ocular imaging. Nguyen et al. administrated PEG-capped AuNPs 20 nm in size into the rabbits as intravenously. These AuNPs showed high PAI and OCT contrast. When the AuNPs circulated into the choroidal and retinal vessels in the living rabbits, the photoacoustic signal of the blood vessels was increased by 82%. The administrated AuNPs provide the possibility for blood vessels detection using photoacoustic microscopy. As well, the AuNPs facilitated visualizing retinal neovascularization and monitoring dynamic changes which happen as a consequence of retinal vein occlusion in the living rabbits [66].

The in vivo photothermal OCT in the eye, was studied for both melanin and gold nanorods as endogenous and exogenous absorbers, respectively [67]. This technique is an OCT-based practical approach generated in a sample for absorbers detection. Albino and pigmented mice were applied to isolate the photothermal signal from the melanin in the retina. Following the systemic administration of gold nanorods to examine their passive accumulation in the retina, the pigmented mouse with laser induced CNV lesions were also observed. This study has shown the combining ability of the PT-OCT technique with gold nanorods to image the distribution of both exogenous and endogenous absorbers in mice eyes. Tzameret et al. assessed the in vivo monitoring with MRI and also the long term protection of IO/HSA NP delivery into the suprachoroid of a rat retinalmodel [68]. In another study, Jaidev et al. evaluated the effectiveness of fluorescent iron oxide NPs against RB cell imaging [69]. Sulforhodamine B was absorbed on the albumin over oleic acid-coated iron oxide NPs. In the MRI research, nanomaterials show a big negative contrast with normal cells and non-cytotoxic cancer cells, proposing their bioavailability. Up to now, iron oxide NPs have been the most utilized NPs in MRIs. The coating procedure could reduce specific toxicity and stability issues [70].

In conclusion, the development of specific and sensitive approaches or tools for the diagnosis of ocular diseases can be useful for world health. Simple and inexpensive approaches that yet have great accuracy; can be developed worldwide. For detection of ocular diseases, ranibizumab-loaded double imaging and therapeutic (S-PEG-ICG-RGD-RBZ) NPs, [56] exhibited the best performance, with regard to good biocompatibility, without any cell dead, cellular toxicity, genotoxicity or apoptosis of developed nanoplatform, however, nanodiscs and gold nanorods reported in this section displayed favorable performance for early detection of diabetic retinopathy regarding high specificity and sensitivity.

### The application of nanomaterials for the treatment of ocular diseases

**Fig. 5:**
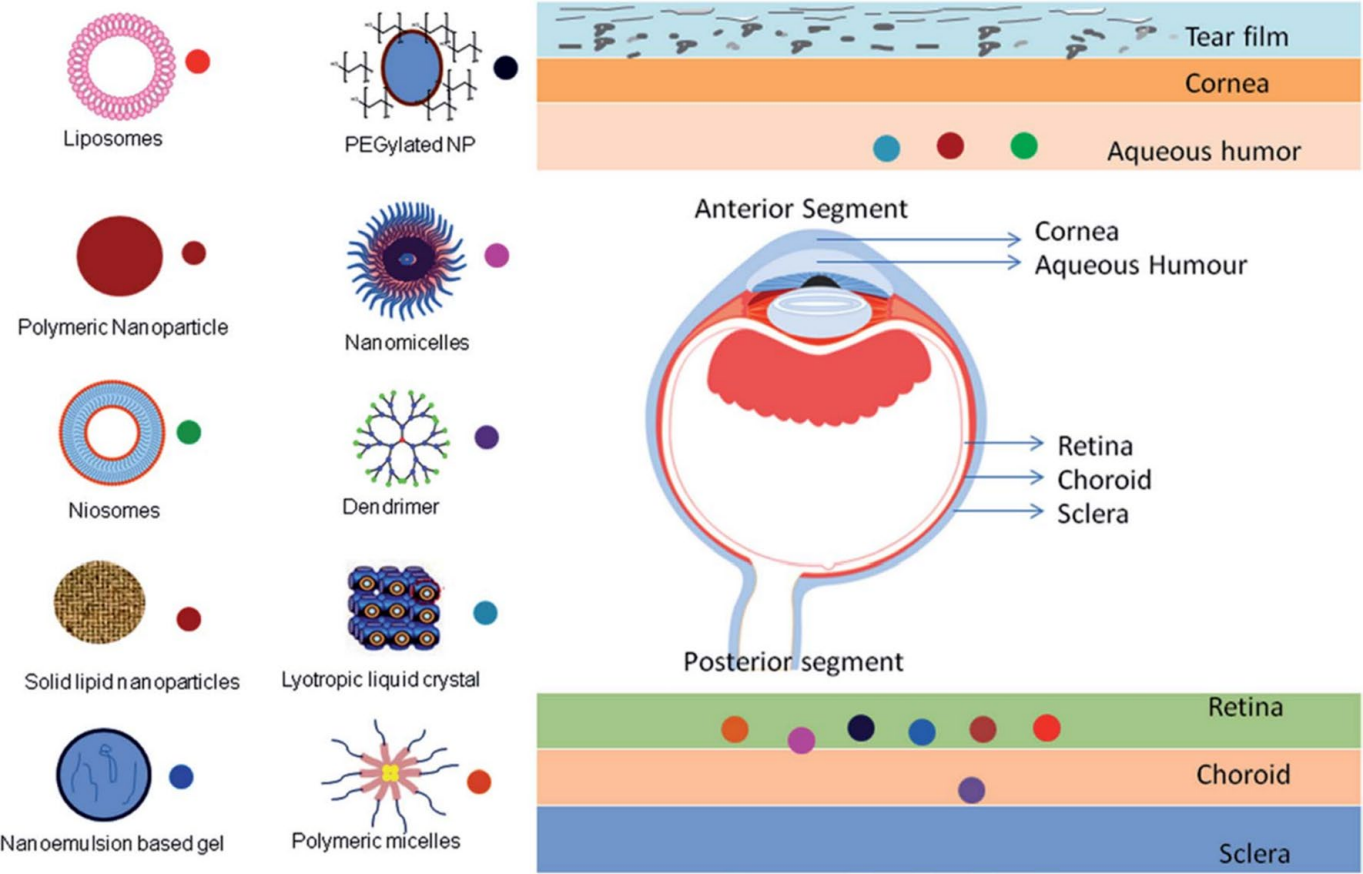
Targeting capability of various nanocarrier platforms. The penetration of nanodrugs through the ophthalmic barrier on topical administration for ocular disease therapy. The symbols next to the nanocarriers show the penetration or targeting ability of the related nanocarriers [3]

Due to their nanoscale size and surface characteristics, nanocarriers have the possibility to overwhelm the ocular barriers and could deliver drugs at the interested site. Various nanocarrier systems and their targeting capability are given in Fig. 5.

### Magnetic-based materials for the treatment of ocular diseases

Magnetic nanoparticles (MNPs) are different from the other nanocarriers owing to the magnetic features that make them unique for drug delivery [71]. Drug molecules could be conjugated to the MNPs shell to be introduced into the body and be concentrated in a local region due to the impact of an external magnetic field. Due to the great surface/volume ratio, it provides many chemically active sites for conjugation of the biomolecule [72]. It enhances the circulation time of drugs into the body and also the possibility of getting to the target site. Moreover, the functionalized MNPs could act as hyperthermia agents, offering an extra therapeutic impact since enhancing temperature in a special site causes cancer cell death without changing normal cells [73, 74]. Also, MNPs could be easily visualized by MRI by the use of an external magnetic field [75]. The most common MNPs applied in biomedical uses are maghemite (ɣFe2O3) and magnetite (Fe3O4) owing to their excellent stability and biocompatibility [76]. Besides, gold, silica, and silver NPs are among the MNPs that have a critical role in the diagnosis and treatment of many diseases [77]. It has been shown that the physicochemical features of MNP affect cellular responses such as cytotoxicity and internalization rate [78]. It is vital to predict all these parameters in the NPs synthesis to get the maximum therapeutic effects [73].

### Magnetic iron oxide-based material for applications in ocular treatments

Specifically, iron oxide NPs offer an auspicious drug delivery system due to their biodegradability and non-toxicity [79–81]. Additionally, owing to the high iron content they could be detected in vivo using MRI [82, 83]. Moreover, various MNPs have FDA-approved for medical purposes, mainly MRI. In the following, we will review the recently published studies of magnetic iron oxide-based material for applications in ocular treatments.

Yanai et al. utilized superparamagnetic iron oxide NPs to magnetize rat mesenchymal stem cells (MSCs) for delivery of cells to the diseased region in the dystrophic retina. Fluid- MAG-D-treated MSCs were intravitreally administrated in a retinal degenerative transgenic rat. The findings exhibited that delivery of magnetic MSC to the retina enhanced tenfold in comparison with normal intravitreal administrated cells. Besides, magnetic NPs therapy together with orbital magnet resulted in considerably greater levels of anti-inflammatory molecule hepatocyte growth factor and IL-10 in the retina. These results proposed that this method might offer optimal benefit in the outer retinal disorders such as AMD that controlled delivery to the focal cells is needed because it could deliver a greater drug load to the target site and result in therapeutically beneficial biochemical changes in the dystrophic retina [84].

Giannaccini et al. have utilized magnetic iron oxide as intraocular delivery for targeting the RPE layer. They utilized MNPs as nanocarriers for ophthalmic drug delivery through intraocular administration in Xenopus embryos, which was able to target the RPE layer and was stable for weeks. Furthermore, the distribution and localization of MNPs in the RPE layer were not dependent on the physicochemical features of particles [85].

Seongtae Bae et al. developed magnetically softened iron oxide (MSIO) nanofluid, PEGylated Fe2O4 for local induction of heat shock proteins (HSPs) 72 in retinal ganglion cells for eye neuroprotection. The RGCs cultured with MSIO nanofluid effectively provoked the induction of HSPs 72. Besides, it was fascinatingly seen that systematic control of “AC magnetically-induced heating up rate” attaining a fixed heating temperature of HSPs 72 induction permitted to get increased induction efficacy at the slowest AC heating up rate throughout MNFH. Along with in vitro experimental confirmation, the investigations of MSIO infusion performance with animal models and a recently produced magnetic coil system exhibited that MSIO has hopeful potential for heat-mediated HSPs agents for managing glaucoma in the future [86].

**Fig. 6:**
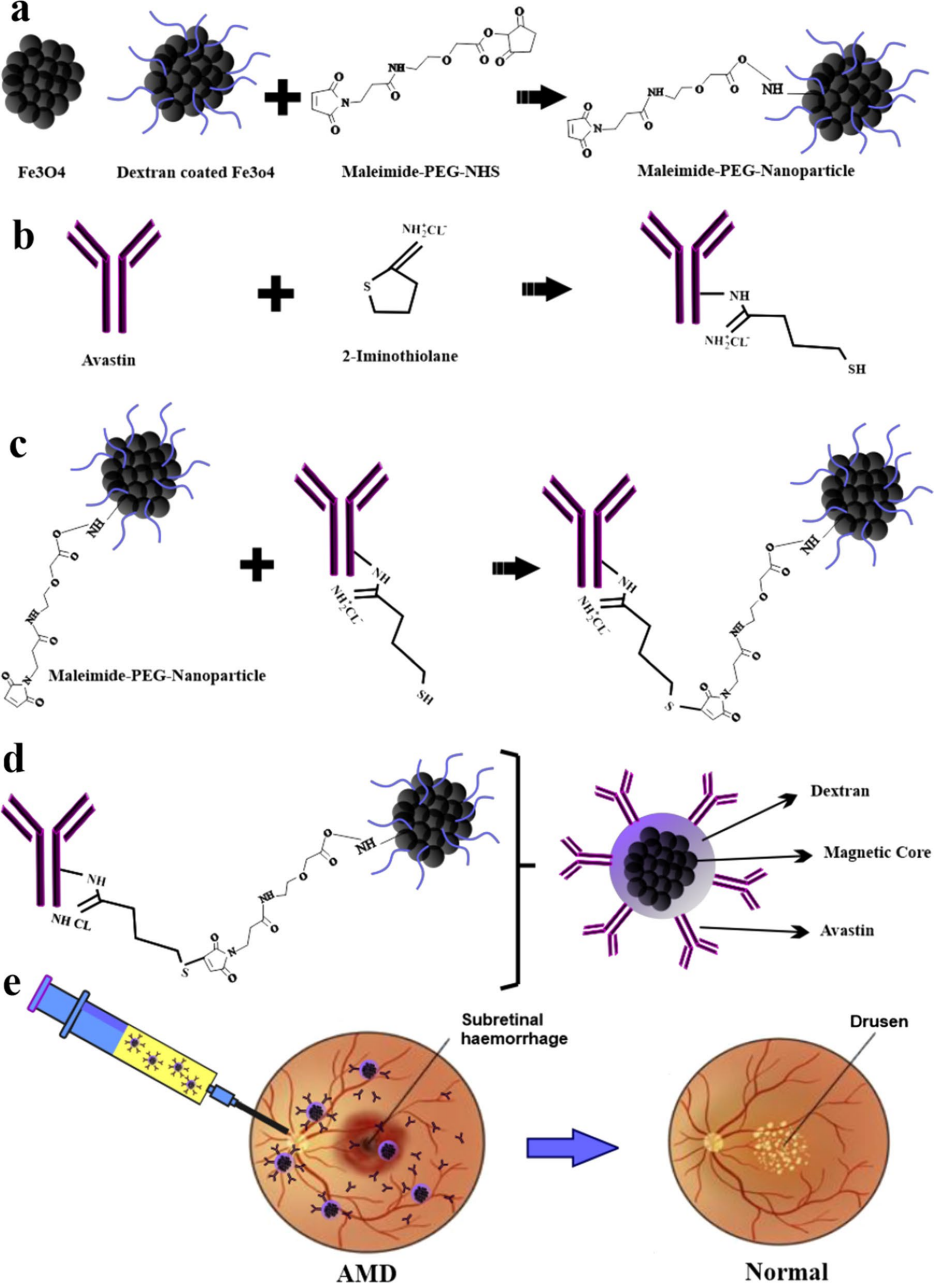
Designing Fe3O4–Avastin nanocomposite as a potential drug for AMD therapy. **a** Preparation and dextran coating of iron oxide NPs **b** Thiolation of Avastin **c**, **d** Avastin loading on the surface of Fe3O4 NPs **e** Intravitreal injection of Fe3O4–Avastin nanocomposite for AMD Therapy [87]

Mehrzad Zargarzadeh et al. have offered a novel drug delivery system by Avastin–Fe3O4 nanocomposites produced via a coprecipitation approach to overcome various difficulties of common therapies for AMD (Fig. 6). The flow cytometry results exhibited that 90.5% of NPs were Avastin loaded. This new approach could be substituted with common therapies due to the long-term release of Avastin rather than numerous injections and decreasing the adverse effects because of the high concentration of Avastin in the posterior eye segment [87].

Yan et al. have developed a successful drug career with engineered Fe3O4 NPs as a facile platform for delivery of ranibizumab in AMD therapy. The results of this study showed that Fe3O4/PEG-PLGA polymer nanomaterials play an effective role in inhibiting tube formation in the Matrigel-based assay approach with human umbilical vein endothelial cells. The evaluations also showed that ranibizumab-treated nanomaterials do not disrupt cell proliferation and the results cannot show any considerable differences in human endothelial cells. The results illustrate that Fe3O4/PEG-PLGA nanomaterials might be very promising as a new formulation for neovascular AMD therapy [88].

In a recently published study, Adi Tzameret et al. assessed the long-term safety and capability to track the Fe3O4 NPs coated with human serum albumin injected into the SCS of a retinal degeneration rat model. In this study, 20-nm IO/ HSA NPs were administrated via suprachoroidal injection into the right eyes of twenty-five RCS pigmented rats, and the left eye, which was considered a control, was not injected. The histological results showed that IO/HSA NPs are detectable in the back segment of the eyes 6 weeks after injection. Also, MRI results exhibited that these NPs are detectable up to 30 weeks. No considerable differences in the retinal function and structure were detected among injected and noninjected eyes [68].

Demirci et al. revealed that IONPs may act as effective therapeutic nano-heaters able to target drug delivery in the eye via intravitreal injection and the use of an external magnetic field for the retinoblastoma treatment. The applied IONPs were tested in the Y79 retinoblastoma cell line, leading to the killing of retinoblastoma cancer cells through the activation of the apoptotic pathway [89].

Recently, Bassetto et al. developed a topical, noninvasive, and magnetically aided delivery system that employs the capability of Fe3O4 NPs to load valproic acid and guanabenz with anti-unfolded protein response features towards the retina. Using MRI, they indicated the existence of Fe3O4 NPs in the retina of Barded-Biedl syndrome wild-type mouse, and their photoreceptor localization was confirmed by transmission electron microscopy (TEM). This technology expands the use of small molecule drugs for the treatment of a wide range of retinal degenerations and other ophthalmic disorders [90].

To date, various magnetic iron oxide-based materials have been applied for the treatment of ocular diseases. With respect to the analytical performance of the reported magnetic iron oxide-based materials in this section, the nanoplatform developed by Adi Tzameret et al. exhibited the best performance due to good safety of suprachoroidal administration of Fe3O4 NPs and capability for long-term detection of the NPs coated with human serum albumin injected into the SCS of a retinal degeneration rat model [68]. Besides, the nanomaterial designed by Seongtae Bae et al. can be a great example of magnetic iron oxide-based materials and has hopeful potential for heat-mediated HSPs agents for managing glaucoma in the future [86].

### Magnetic gold-based materials for applications in ocular treatments

Gold nanoparticles (GNPs) are considered auspicious platforms for biosensing and therapy due to their low cytotoxicity, and exceptional optical and very tunable properties [91, 92]. Recent examples of gold-based materials for applications in ocular treatments are reviewed below.

Cho et al. have utilized topically administered gold NPs for inhibition of corneal neovascularization in mice. They showed that the neovascularized area in the gold NP-treated group was decreased by 39%, and also the expression of VEGFR-2 was diminished, which prevented the inflammation [93].

Salem et al. have examined a formulation of liposomal flucytosine capped with gold NPs for increasing intraocular permeation and therapeutic effectiveness. The finding of this study showed that the topical administration of flucytosine capped with gold NPs could be successfully used for the treatment of the experimental C. Albicans cornea infection [94].

Akihiro Hoshikawa et al. developed ranibizumab/PEG-conjugated gold NPs (AuNPs) as a new platform for the delivery of ranibizumab. Ranibizumab/PEG-conjugated AuNPs were successfully produced. It was shown that ranibizumab/PEG-conjugated AuNPs successfully repressed the tube formation of human umbilical vein endothelial cells based on Matrigel in vitro. Surprisingly, PEG-conjugated AuNPs without ranibizumab prevented the tube formation. The ranibizumab/PEG-conjugated AuNPs don’t disturb cell proliferation in human cells. The results propose that ranibizumab/PEG-conjugated AuNPs could be utilized as a new formulation against angiogenesis-associated disorders such as AMD [95].

Maulvi et al. studied the effect of GNPs on loading and its release from the contact lens using the soaking approach. In the first method, GNPs were loaded into the timolol soaking solution, and in the second method, GNPs were included in the contact lenses in the course of fabrication. The finding showed a considerable increase in the drug uptake and loading capability of therapeutic contact lenses with the presence of GNPs in the solution. The in vivo evaluations exposed that these contact lenses enhanced the deposition of drugs inside the intraocular tissues, thereby extending the IOP-lowering time [96].

It has been reported that the resveratrol-coated gold NPs administrated in streptozotocin-induced diabetic rats can offer a protective impact in the case of diabetic retinopathy for three months. This protective impact of gold NPs can assist to recover the balance of the inhibitors and stimulators of the angiogenesis procedure via the inhibitory impacts of the ERK1/2 pathway and expression of NF-κB (nuclear factor kappa B), which can diminish permeability and inflammation of the blood-retinal barrier (BRB) in the diabetic rats. Besides, there was a considerable reduction in all the retinal mRNA expressions of VEGF-1, interleukin 6 (IL-6), and tumor necrosis factor-alpha (TNFα) [97].

It was found that plasmid DNA-wrapped gold NPs can be effectively internalized in ARPE-19 cells with great transfection efficacy. These findings were verified with the initial expression of a reporter gene that was observed at 16-h after transfection. The findings illustrate a possibly effective gene delivery route to RPE cells by gold NPs; however, they suggest that the mechanism of cell interaction with gold NPs should be more perceived to enhance the transfection effectiveness of these particles and evade the autophagic pathways of the particles to ensure the effect of stable gene expression in their system [98].

Gold-coated MNPs were produced and coated with ranibizumab as an ophthalmic drug delivery system. Ranibizumab conjugation on NPs was done by the physical adsorption approach. The ranibizumab amount on the NPs surface was determined using thermogravimetric assay. In vitro release analyzes showed approximately 60% of antibodies were released in the initial 30 min. Also, the activity of antibodies following release analyses was verified with ELISA assay. Nontoxicity of gold-coated Fe3O4 particles was shown with MTT. The results revealed that the antibody-conjugated MNPs can be a possible treatment system for eye disorders [99].

**Fig. 7:**
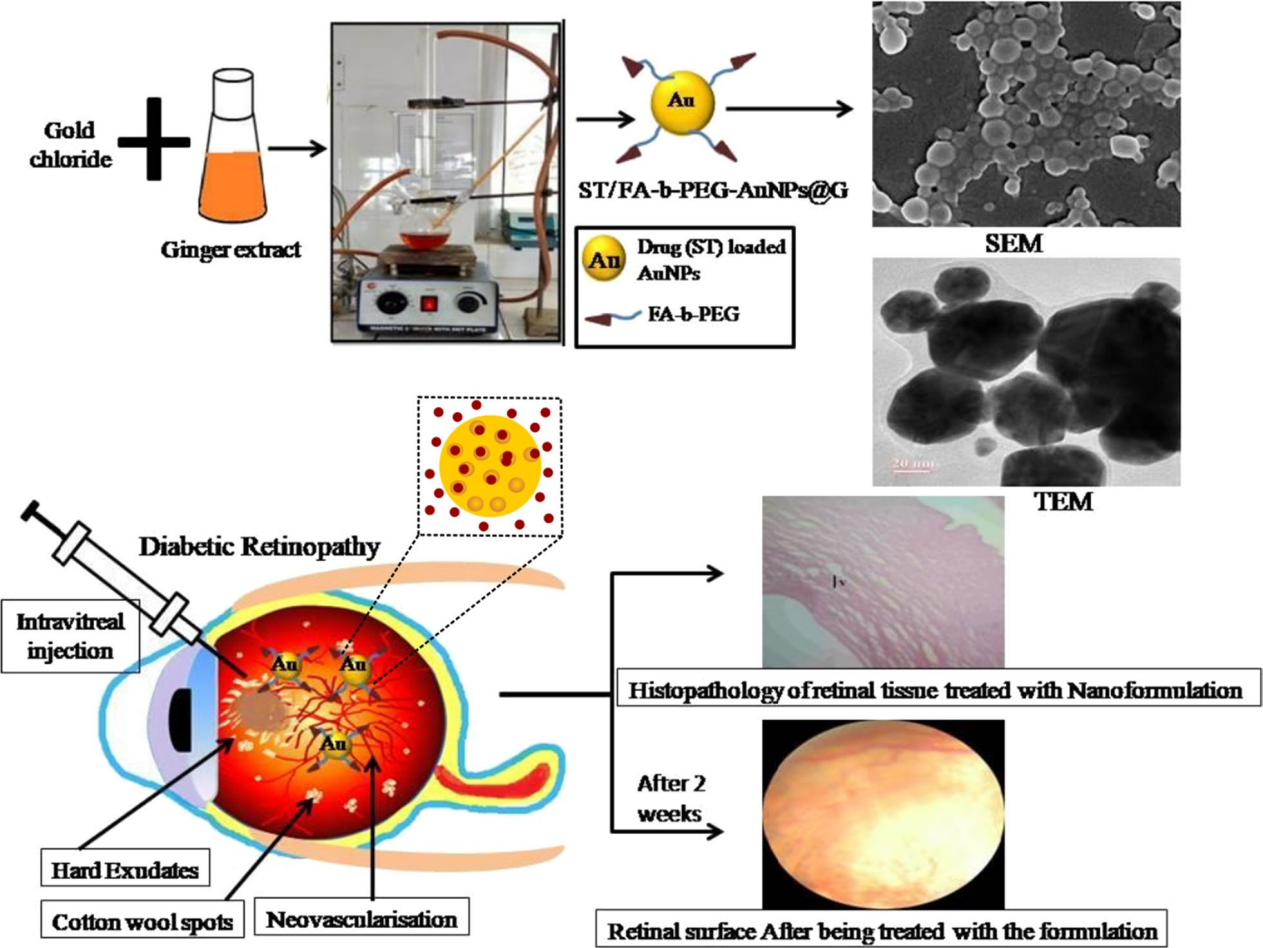
Production of AuNPs using green synthesis for diabetic retinopathy therapy [100]

The outstanding features of AuNPs make them a good carrier to carry and release drugs at a specific site in a controlled mode. Recently, VivekDave et al. produced AuNPs using green synthesis for diabetic retinopathy therapy (Fig. 7). These NPs have been modified with FA-b-PEG co-polymer for the directed delivery of Sorafenib tosylate. This chosen drug acts on VEGF-receptors in the retinal neovascularisation region. Histopathological analyses and fundus photography has been utilized for the in vivo characterization of the prepared dosage form and it has been found that it could be a successful therapy for diabetic retinopathy [100].

Recently, P.S. Apaolaza et al. used hyaluronan to enhance the mobility of NPs and target them to HA receptors expressed in various eye cells. Combining HA and gold increased the stability of the whole carrier and their distribution across ocular tissues and barriers to reaching the retina. Furthermore, in vitro and ex vivo assays showed the anti-angiogenic effect of gold NPs as inhibitors of AGEs mediated retinal pigment epithelial cell death and neovascularization. They revealed that NP conjugation with HA increases the distribution and stability of gold NP owning to a particular CD44 receptor interaction. The ability of HA-gold NPs to distribute via the vitreous humor and their avidity for the deeper retinal layers ex vivo, propose that HA-gold NPs are auspicious delivery platforms for ocular neovascularization-related diseases [101]. Following intracameral administration, the surface/size-dependent distribution of the NPs was investigated after ex vivo perfusion of porcine eyes. Furthermore, to assess the effective cellular uptake of the NPs, in vitro cell culture experiments were done. HA-AuNPs showed excellent colloidal stability. Even after ex vivo use in porcine eyes, the HA coating inhibited NPs aggregation within the trabecular meshwork. NPs with a 120 nm diameter showed the greatest volume-based accumulation in the trabecular meshwork. Off-target tissues in the anterior chamber showed an outstandingly low gold content. The results of this research are especially significant for NPs with encapsulated anti-glaucoma drugs owning to a greater particle volume that might be accompanied by a better drug load [102].

In conclusion, among the magnetic gold-based materials presented in this section, nanoplatform synthesized by Maulvi et al. showed the best performance due to its non-cytotoxic, non-immunogenic and biocompatible nature of GNPs and illustrated notable enhancement in the deposition of drug with the GNPs-laden contact lenses in the conjunctiva and ciliary muscle [96]. As well as, resveratrol-coated gold NPs reported by Dong et al. displayed great performance in the treatment of diabetic retinopathy with respect to the protective effect of gold NPs on streptozotocin injected diabetic rats that can aid in redeveloping the balance between the stimulators and inhibitors of angiogenesis [97].

### Magnetic silica-based materials for applications in ocular treatments

Mesoporous silica nanoparticles (MSNs) have presented great capacity in drug delivery owing to great stability, high surface area and pore volume, and changeable pore diameter [103]. The research interest of MSNs is due to the easy production and the adaptability of the drug combination and surface functionalization; actually, they could integrate inorganic material inside or on the structure surface and an extensive variety of molecules could be linked to the surface of silica due to flexible silane chemistry. Also, SiNPs are stable and biocompatible carriers with extended blood circulation because of their hydrophilic surface [104–106]. Here, recent examples of silica-based magnetic materials for applications in ocular treatments are given.

Park et al. performed a study to assess the cytotoxicity of various sizes of silica NPs (SiNPs) on the surface of ocular cells such as human corneal epithelial cells (HCECs). The findings of this study proposed that cellular autophagy pathway activated with the adding SiNPs without any considerable cytotoxicity in the cultured HCECs [107].

Pilocarpine-encapsulated MSNs gelatin-covered, prepared by Liao et al. let progressive and continuous drug leakage from their porous structure throughout the slow gelatin degradation and attained 21 days reduction of IOP following one intracameral injection. They showed that the matrix metalloproteinase-2 expression in the anterior chamber causes gelatin degradation, which produces a moderately acidic environment that permits pilocarpine release from G-MSN and decreases IOP. Animal and cellular studies results proved the controlled release of pilocarpine, and its effect on the reduction of IOP. So developed nanocarriers could be possibly beneficial in glaucoma therapy [108].

Brimonidine eye drops showed restricted efficacy in glaucoma therapy owing to their fast elimination from preocular space. To overcome this problem, Kim et al. performed a study on the delivery of brimonidine with amino-functionalized mesoporous silica (AMS) particles such that AMS particles adhere to the mucous layer and let high preocular residence time. When topically administered into the rabbit's eyes, BMD-AMS remained for up to 12 h in preocular space. The findings showed that the duration of IOP reduction and the area under the drug concentration in the aqueous humor-time curve in BMD-AMS were twice as many compared to Alphagan P. The results exhibited the enhancement in ophthalmic bioavailability of brimonidine with BMD-AMS [109].

**Fig. 8:**
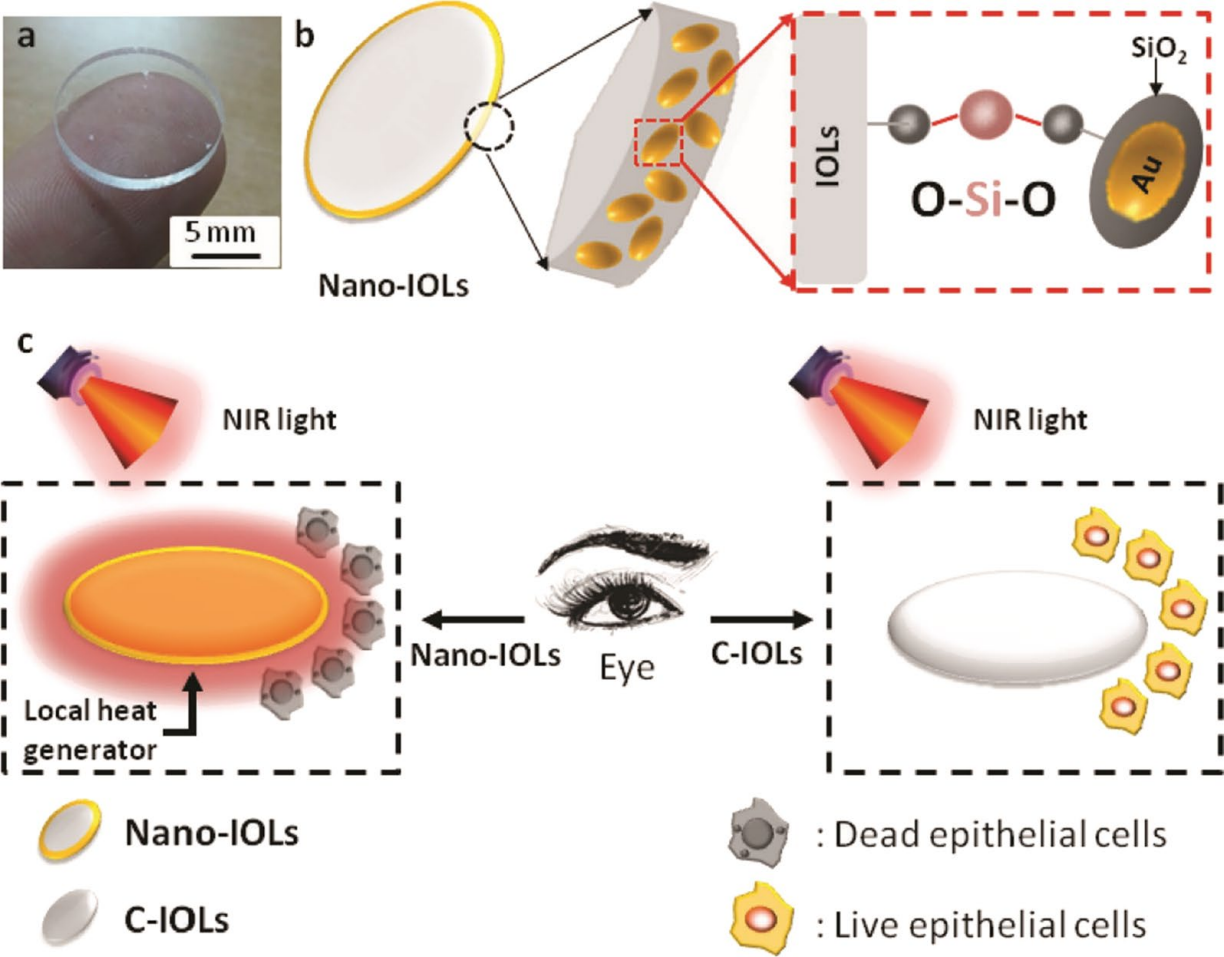
The graphical representation of Nano-IOLs to prevent PCO; **a** The digital figure; **b** Nano-IOLs with nanostructured Au@SiO2 external rim; **c** The mechanism of action of Nano-IOLs for PCO prevention. The Nano-IOLs implanted in the cataract rabbit eyes prevent the lens fibrosis via area-confined photothermal therapy under the Near-infrared irradiation. Adapted with permission from [110]

Lin et al. utilized, a nanostructured photothermal ring integrated intraocular lens (Nano-IOLs), in which the rim of C-IOLs (Commercial IOLs) is decorated with Au@SiO2 (silica-coated Au nanorods) that could successfully avoid posterior capsule opacification (PCO) that happens following cataract surgery (Fig. 8). The Nano-IOLs can eliminate the residual lens epithelial cells (LECs) around Nano-IOLs under mild laser therapy and block the formation of disordered LECs fibrosis, which finally results in vision loss. In vivo studies show that using Nano-IOLs, PCO incidence is about 30%-40% in rabbit models, which is considerably lower than the control group that was treated with C-IOLs 30 days post-surgery. The findings showed that spatial controllability of photothermal effect of nanomaterials can offer an exceptional method to intervene the PCO-mediated vision loss [110].

Jin Yang et al. used CeCl3@mSiO2 NPs with an approximate size of 87.6 nm for a streptozotocin-mediated diabetic cataract rat model with intraperitoneal administration. The results of this study demonstrated that CeCl3@mSiO2 effectively alleviates diabetic cataract progression. Along with the antioxidant effect of CeCl3@mSiO2 in vitro, injection of CeCl3@mSiO2 considerably abolished hyperglycemia-induced upregulation of advanced glycation end products, protein carbonylation, and lipid peroxidation in the animal lens [111].

Notably, MSNs can successfully enter the cornea. Hu et al. produced SNP-loaded MSNs as eye drops for sustained release of NO at the Schlemm’s canal area and trabecular meshwork. In vivo assays showed extended diminution of IOP from 3 to 48 h through the MSNs involvement, with just 1/40 of the dose of SNP solution [112]. However, the possible toxic effects of SNP on the common outflow tissue has found that the continuous usage of this NO donor may lead to protein nitration. Moreover, the involvement of magnesium hydroxide NPs has led to increased corneal permeability and IOP-diminishing efficacy of hydrophilic antiglaucoma drugs [113, 114].

In the conclusion of this section, we found that the Nano-IOLs platform designed by Lin et al. is a highly efficient approach for intervening in the PCO-mediated vision loss regarding its great biocompatibility and also exceptional area-confined photothermal effect [110]. However, CeCl3@mSiO2 NPs developed by Jin Yang et al. showed favorable performance for effective alleviation of diabetic cataract progression due to its biocompatible and biodegradable features and reduction of antioxidant enzyme activity, lipid peroxidation, and also scavenges free radicals [111].

### Polymeric-based materials for the treatment of ocular diseases

Because of their mucosal adhesive properties, polymers are extensively used as drug carriers in ocular drug delivery. This polymer^,^ s property increases the shelf life of the drug in the cornea and conjunctival epithelium, facilitating the reduction of the rapid clearance of the drug from the eye, which is frequently experienced with topical ophthalmic formulations [115]. Some polymers have been presented to be stimuli-responsive, permitting them to release an active ingredient after a change in conditions, for example, changes in pH, temperature, and pressure. Similarly, smart polymers have been employed in situ as gelling systems. A further advantage to the usage of some polymers is their biodegradability such that they are broken down by the body into nontoxic elements. After breaking the polymer, the drug is released, leading to constant drug release profiles. Biodegradability eliminates the need for manual removal of the drug delivery system, which is particularly significant in the development of ocular implants [116, 117].

Recently, some polymers such as microspheres [118, 119], micelles [120], and hydrogels [121] have been studied for utilization as a carrier for sustained drug delivery in the eye. Among these, hydrogels have obtained considerable attention because of their versatile features [121, 122]. These polymeric materials could absorb high quantities of water and have the ability to modify their physical characteristics, e. g. transitioning from solution-to-gel, or gel-to-solution, in response to external stimuli, for example, temperature, pH, magnetic field, and ionic strength [123, 124]. In the following, two classes of polymeric-based materials for the treatment of ocular diseases will be discussed.

### Lipid-based materials for the treatment of ocular diseases

**Fig. 9:**
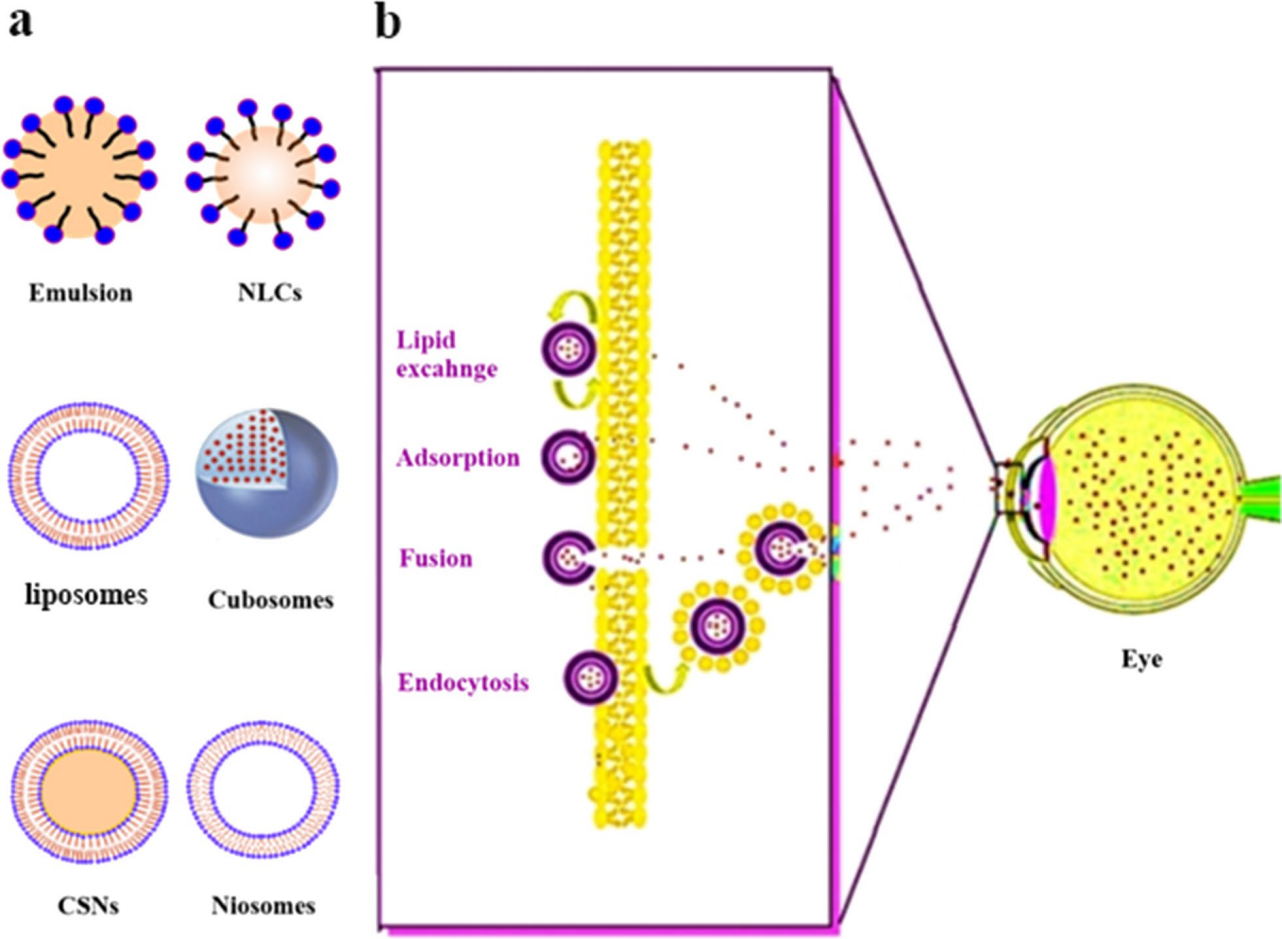
**a** Different lipid-based nanocarriers **b** The procedure followed by lipid-based nanocarriers overwhelming the ocular barrier [70]. NLCs: Nanostructured lipid carriers; CSNs: Core/shell nanoparticles

Solid lipid nanoparticles (SLNs) with an average size of 10-1000 nm are made of solid lipids whose core lipid matrix can dissolve hydrophobic parts. Site-specific drug delivery, excellent biocompatibility, high stability, high drug entrapment with customizable particle size, high surface-to-volume ratio, low manufacturing cost, etc. make SLNs attractive carriers for ophthalmic drug delivery [125, 126]. The hydrophobic system of lipid molecules interacts with water, causing self-assembly of lipids through liposome formation. Figure 9 shows some different lipid-based nanocarriers (Fig. 9a) and the procedure followed by lipid-based nanocarriers overwhelming the ocular barrier (Fig. 9b). Recent examples of lipid-based materials for the treatment of ocular diseases are reviewed below.

Balguri et al. investigated the therapeutic effect of SLNs and nanostructured lipid carriers (NLCs) of indomethacin by topical use. This NPs system presents a greater efficacy for drug loading and entrapment capacity. These lipid-based nanoparticulate systems could be employed as viable carriers in ocular infection therapy and can facilitate the delivery of drugs into the anterior and posterior ocular tissue segments [127, 128].

Natamycin (NAT) as the first FDA-approved drug used to treat fungal keratitis suffers from poor corneal penetration, which restricts its effectiveness in the treatment of deep keratitis. Amoabediny, Ghasem et al. produced NAT-SLNs to achieve sustained drug release and enhanced permeation into the corneal. It was found that NAT-SLNs are nonirritating to corneal tissue. This formulation showed an extended drug release rate that increased corneal penetration, and enhanced antifungal activity with no cytotoxic effects on corneal tissues. Therefore, NAT-SLNs offer an auspicious ophthalmic drug delivery system for treating deep corneal keratitis [129].

Zhenjie Mo et al. produced a new Bimatoprost (BIM) NPs-loaded pH-sensitive in-situ gel for glaucoma therapy. In-vitro and ex-vivo assays of BIM-SLN4, SLN-ISG3 exhibited drug release for a long time. HET-CAM test exposed that this nanoformulation is nonirritant and therefore could be well-tolerated when used in the eye. Also, histopathological investigations did not show any tissue damage signs. So, SLN-ISG could offer better glaucoma management [130].

Arpita Bhattacharjee et al. developed amphotericin B (AmB)-loaded PEGylated-NLC and investigated its ocular distribution ability after topical use. AmBPEG2K-NLC cytotoxicity was examined in human RPE cells. In vivo ocular biodistribution of AmB was assessed in rabbits, after topical use of PEGylated-NLCs or marketed AmB preparations. PEGylation considerably inhibited AmB leaching and improved the drug loading. No toxicity was observed up to the highest concentration of AmBPEG2K-NLC. After topical application, AmB was recognized in all the ocular tissues and statistically significant (p > 0.05) difference was not seen between the formulations examined [131].

The formation of new blood vessels is straightly associated with the ocular disease’s occurrence. Anti-angiogenic medicines could be employed in the treatment of eye disorders. In a study by Shuai Shi et al. axitinib was loaded through the amphiphilic co-polymer MPEG-PCL, increasing its ability to dispersion in water. Axitinib-loaded micelles displayed low toxicity in concentration gradient evaluates. Moreover, numerous doses with scratch tests proved that axitinib did not have any considerable impact on the migration of normal cells, and the results of the biosafety assay exhibited great cell biocompatibility. Following the development of the corneal neovascularization model after an alkali burn in rats, the anti-angiogenic effectiveness was investigated, with the positive control (dexamethasone). The results of this study exhibited that axitinib-loaded micelles had anti-angiogenic impacts without noticeable tissue toxicity. It is concluded that axitinib could be utilized in the treatment of ophthalmic neovascular disorders via nanocrystallization [132].

Statins are extensively recommended for cardiovascular disorders, as well, it has been used to treat AMD. However, the blood-aqueous barrier and low bioavailability can reduce the ocular statins concentration after oral administration. Monika Yadav et al offer local use of atorvastatin (ATS) loaded into SLNs, as self-administrable eye drops. High molecular weight, instability, and insolubility of ATS, and whether SLNs reach the back of the eye were the challenges to be faced. ATS-SLNs that were produced by appropriate constituents, quality based on design method, and scalable hot high-pressure homogenization, were assessed widely for ophthalmic appropriateness. ATS-SLNs were 8 (in aqueous) and 12 (in vitreous humor) times further bioavailable, compared to free ATS. ATS-SLNs showed good ocular safety, 2.5-fold corneal flux, and 13.62-fold stability. Fluorescein-labeled SLNs observed in the eye after using as eye drops offer reliable evidence of efficient delivery. Perinuclear fluorescence in ARPE-19 cells verifies the useful F-SLNs uptake. Extended retention time up to 7 h, was ascribed to the mucus-penetrating feature of ATS-SLNs [133].

Kaichao Song et al. studied the capacity of glycyrrhizin-based self-assembled nanomicelles in ocular topical uses. It was found that DG-THY improves in vitro release and antioxidant activity, and the THY permeability. This ophthalmic solution was well tolerated in the rabbit model. The DG-THY ophthalmic solution showed clear enhancement in the in vivo and ex vivo intraocular THY penetrations as well as reduced minimum inhibitory and bactericidal concentrations. Also, the DG-THY solution considerably alleviated the symptoms of eye infection in the rabbit eyes. Thus, the findings revealed that DG-THY might be an auspicious ocular formulation for the treatment of inflammation-, oxidative stress- and bacteria-related ocular diseases [134].

Besifloxacin hydrochloride (BSF), as a novel ocular antibiotic, suffers from poor water solubility, limiting its therapeutic efficiency. Mirza Salman Baig et al. described a new lipid-based drug delivery system to increase the BSF ocular bioavailability. Cationic nanostructured lipid carriers (CNLC) were formulated and the surfactant hexadecyltrimethylammonium bromide (CTAB) was utilized for optimization of the surface charge of the NPs. The CNLC cell internalization is enhanced when the concentration of CTAB is enhanced in CNLC-BSF. This nanoformulation exposed suitable permeation characteristics throughout the 3D tissue model. The cytotoxicity evaluated using the MTT test exhibited at least 60% cell viability on the conjunctival fibroblast model with the administration of 0.6 mg/mL BSF [135].

An overview of the reported lipid-based materials for the treatment of ocular diseases, revealed that the ATS-SLNs designed by Monika Yadav et al. showed the best performance, as self-administrable eye drops, due to great bioavailability and stability, extended retention time compared to free ATS as well as good ocular safety [133]. However, nanoformulation reported by Zhenjie Mo et al. showed good efficacy for the treatment of glaucoma with regard to long-time drug release and good biosafety [130].

### Polysaccharide-based materials for treatment of ocular diseases

In recent decades, extensive research efforts have been made to develop polysaccharide-based biomaterials which could be facilely accepted by tissues and cells [136]. In comparison with synthetic nanomaterials, polysaccharide-based nanocarriers show superior efficiency in respect of the retention of drug and ocular penetrability through the permeation of mucin chains [137–139]. For instance, chitosan, as a kind of polysaccharide with positive charge and linear structure, could closely combine with the negatively charged conjunctiva and cornea. Therefore, this electrostatic interaction increases the permeation ability and extends the residence time [140]. Furthermore, polysaccharides are usually present in the eye, e. g. hyaluronic acid, one of the main ingredients of the vitreous. Developing research has shown the polysaccharides' biocompatibility and their derivative nanomaterials for ocular delivery [141–144]. Evidence obtained shows their good tolerance, biosafety, and greater bioavailability, hence, polysaccharide-based nanocarriers have obtained much attention for medical uses [145]. In the following, we will review the recently published studies of polysaccharide-based materials for ocular disease therapy.

In a study by Dan Liu et al. to increase the ophthalmic bioavailability of moxifloxacin hydrochloride, hyaluronic acid-modified lipid polymer hybrid NPs (HA-LCS-NPs) have been prepared. To evaluate the mean retention time, area below the curve of HA-LCS-NPs, and the corneal penetration in rabbits, in vitro and in vivo assessments were done. The in vivo results indicated that mean retention time is up to 6.74-fold and the area below the curve of HA-LCS-NPs is 4.29-fold greater than those on the market. Also, the in vitro results indicated that the penetrability of HA-LCS-NPs was enhanced by 3.29-fold than commercial products. Additionally, compared to common formulations, the ex vivo results of fluorescence imaging indicated that the fluorescence strength was greater in the conjunctiva and cornea following HA-LCS-NPs administration. Ultimately, an eye irritation test showed that HA-LCS-NPs exhibited outstanding eye tolerance. It is concluded that the hyaluronic acid-modified lipid polymer hybrid NPs with multifunctional features may be a hopeful ophthalmic drug delivery system for long-term precorneal retention, enhanced cornea penetrability, and improved ocular bioavailability [146].

Neeraj Mittal et al. designed timolol maleate (TML)-loaded polymeric NPs for ophthalmic delivery by ionic gelation approach. The formulated NPs exposed significant bioadhesive ability and showed sustained drug release in experimental evaluations. The ex vivo transcorneal penetration research showed greater corneal TML permeation comparing marketed eye drops. Also, the confocal scanning laser microscopy (CSLM) studies proved the NP's ability to permeate into the cornea. The histopathological investigations exposed the corneal biocompatibility of NPs. Compared with common eye drops, the NPs decreased IOP in rabbits for a long time. The results proposed an auspicious role of polymeric NPs for ophthalmic drug delivery in glaucoma therapy [147].

Chaharband et al. produced hyaluronic acid-chitosan nano-polyplexes loaded with siRNA via an ionic gelation technique that can permeate the retina barriers and vitreous. Intravitreal administration tests showed that the nano-polyplexes can attain the back of the rabbits' eyes and successfully diminish the size of laser-mediated neovascularization [148]. As well as, biological macromolecules e. g. peptides may be utilized to develop polysaccharide-based hybrid nanocarriers [149, 150]. Silva et al. synthesized hyaluronic acid-chitosan NPs loaded with erythropoietin. The in vitro penetration assays exhibited fast permeation into porcine conjunctiva followed by the cornea and sclera, without cytotoxicity [151].

Numerous researchers have proposed that some natural polysaccharides, specifically plant-derived ones, have exceptional biological functions for the ocular system. It has been shown that polysaccharides of Lycium barbarum improve dry eye syndrome, avoid oxidative damage in human trabecular meshwork cells, and maintain the retina and ganglion cells' function [150, 152–156].

Recently, resveratrol (RSV), has received much attention as an accepted medicine owing to its potent anti-inflammatory and antioxidant characteristics. Nevertheless, RSV suffers from chemical instability and low aqueous solubility as well as inefficient delivery to the back of the eye. Nano-based technologies have appeared as a potential solution to overcome these drawbacks. Buosi et al. developed nanogels (NG)-based on chitosan (HCS) that cross-linked with sodium tripolyphosphate. Biocompatibility tests showed that nanogels have non-cytotoxic and non-inflammatory effects in human ARPE-19 cells, which creates the outer blood-retinal barrier. Upon cellular internalization, they reported an endo-lysosomal escape of NG, which is critical for effective nanocarriers delivery systems. It is concluded that HCS-based NG might comprise new vehicles for RSV, opening the opportunity for its use in eye disorders [157].

**Fig. 10:**
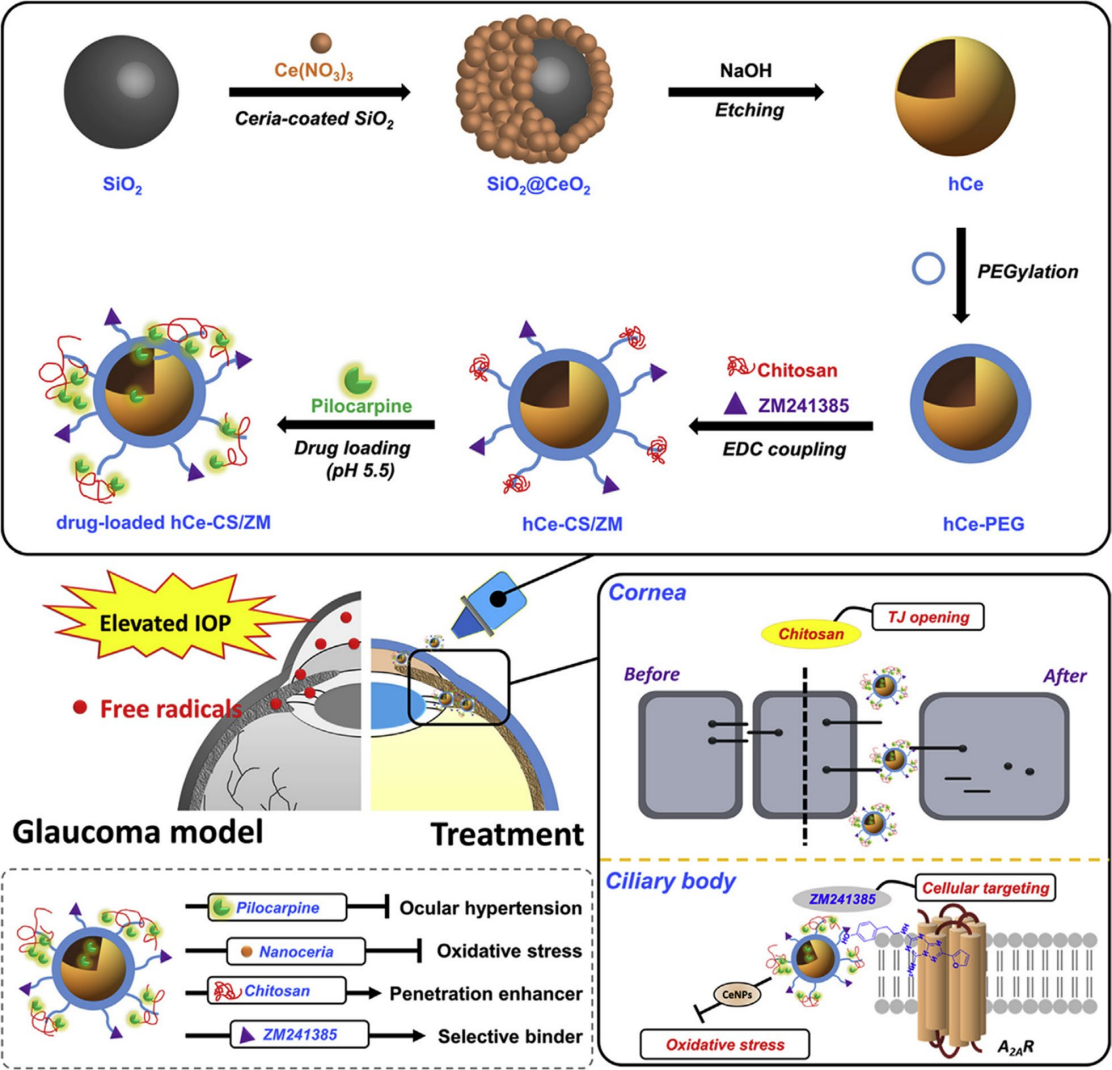
Preparation of nano eye drops and their use for the treatment of glaucoma. Production of hollow ceria NPs and then their dual functionalization with ZM241385/chitosan and also loading with pilocarpine for usage as nano eye drops. Topical delivery of the nano eye drops and their pharmacological/biological functions for opening the tight junctions of corneal epithelium, targeting drug molecules toward the ciliary body tissue, and attenuation of inflammation and oxidative stress for successful treatment of glaucoma [158]

Luo et al. produced bi-functional NPs with improving ZM241385 and chitosan on the surfaces of hollow ceria NPs loaded with pilocarpine. It was found that the hollow structure considerably enhanced the drug residence time. Additionally, ZM241385 and chitosan had the ability to permeate the cornea, whereas ceria provoked anti-inflammatory and antioxidant activities. These NPs showed a 42-fold longer time of reducing the intraocular pressure in comparison with marketed eye drops (Fig. 10) [158].

Jiang et al. designed a nanocarrier based on core–shell structured polysaccharide with a chitosan core and polycaprolactone shell via a 2-step emulsion technique. Thru electrostatic interactions, the chitosan core was filled with bevacizumab. The core–shell particles had a considerably enhanced ability to prolong the release of drugs for three months and have useful outlooks as an anti-VEGF drug in medical use [159].

Recently, Zoratto, Forcina et al. explained the activity of hyaluronan-cholesterol nanogels (NHs) as ophthalmic penetration enhancers. Due to their bioadhesive attributes, NHs tightly interact with the surface corneal epithelial cell, with no permeation into the stroma, improving the transcorneal permeation of loaded drugs. Ex vivo transcorneal penetration assays indicated that the penetration of hydrophilic drugs, loaded in NHs, is considerably increased when compared with the free drug solutions. Besides, the penetration of hydrophobic drugs is greatly reliant on the water solubility of the trapped molecules. The findings propose that this formulation could enhance the ocular bioavailability of the instilled drugs by enhancing their preocular residence time and also simplifying their penetration, therefore opening the way for use of HA-based NHs in treating anterior/posterior eye segment disorders [160].

One of the most important derivatives of chitosan, named carboxymethyl chitosan (CMCTS), with high biodegradability and biocompatibility, is used as a vitreous substitute. In a study conducted by Wang et al. oxidized hyaluronic acid (OHA) was produced as a cross-linking reagent. OHA and CMCTS were utilized to produce self-repairing, biodegradable, biocompatible, and in situ injectable hydrogels as vitreous substitutes. This biocompatible hydrogel with high transparency and controllable swelling properties was injected into the vitreous cavity after vitrectomy and no significant side effects were seen for 90 days after injection in New Zealand Rabbits. In addition, intraocular pressure and retinal position were maintained in the operated eyes. It is concluded that this non-toxic, injectable, and biodegradable hydrogel has immense capacity to develop material for vitreous substitutes [161].

The retinal disease treatment using intravitreal administrations needs regular injection except if drug delivery systems with extended residence time and controlled-release are utilized. Recently, Eva Kicková et al. employed pullulan conjugates of dexamethasone as therapeutic systems for intravitreal injection. They showed that these pullulan-based drug conjugates are safe in rabbit, mouse, and rat eyes, have a long residence time in the vitreous, and are approximately wholly eliminated through the aqueous humor outflow. The findings of this study revealed that pullulan–dexamethasone conjugates might release active and free dexamethasone in the vitreous humor for higher than 16 days, although a big dexamethasone fraction might be deleted from the eye as bound pullulan–dexamethasone [162].

Regarding all reported polysaccharide-based materials for treating eye disorders, it seems that the therapeutic system reported by Eva Kicková and coworkers provides great intravitreal drug delivery systems because they could diminish the frequency of injection and transfer of drugs into retinal tissues. Besides, this nanoplatform showed good biosafety and long residence time in the vitreous [162]. Also, the nanogels-based on chitosan reported by Buosi et al. offered successful nanocarriers delivery systems with respect to non-cytotoxic and non-inflammatory effects in human ARPE-19 cells, opening the good opportunity for its use in ocular disease [157].

## Nano-biomaterials for regenerative ophthalmology

Recently, regenerative medicine is giving promises for reinstating the function of aged and diseased organs and nanotechnology is assisting as a catalyst. In ophthalmology, many kinds of autologous and allogeneic stem cells have been examined for the treatment of certain ophthalmic disorders including AMD, retinitis pigmentosa, glaucoma, diabetic retinopathy, and lens and corneal traumas. The nanomaterials have been used straightly as nano-scaffolds for the stem cells to stimulate proliferation, differentiation, and also their adhesion, or indirectly as vehicles for delivery of some genes, immunosuppressants, cytokines, and tissue growth factors for facilitating cell reprogramming or regeneration of eye tissue [163].

**Fig. 11:**
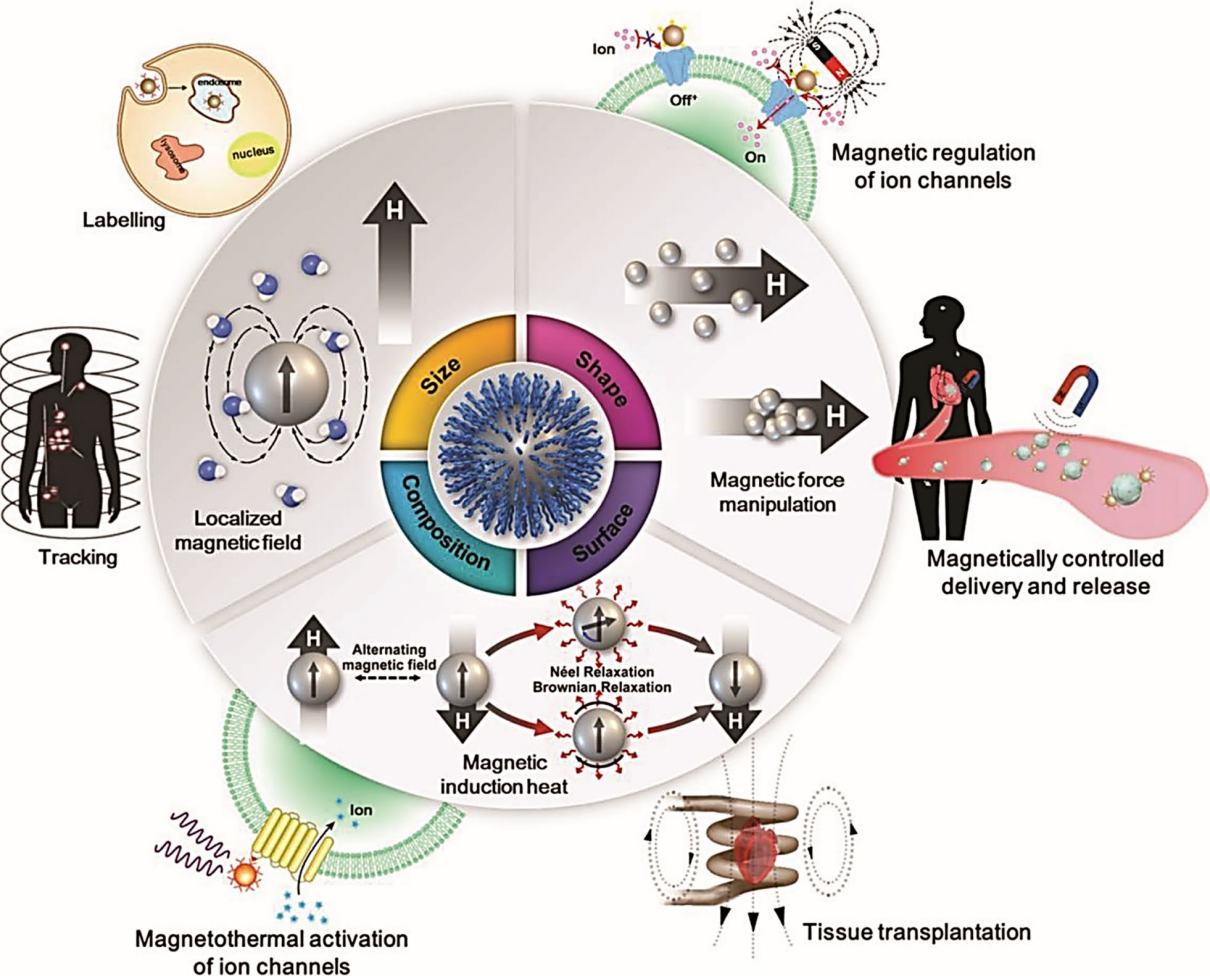
Diagram illustrating how MIONs might be beneficial in the tissue regeneration area. Adapted with permission from [166]

The biocompatibility of different organs with many nanomaterials, for instance, NPs, hybrid nanostructures, and nanowires (NWs) have increased the possibility of their usage in clinical uses, particularly in retinal regeneration [163–166]. Amongst these, NPs such as magnetic iron oxide nanoparticles (MIONPs) and AuNPs are extensively employed in pre-clinical and clinical settings [167–169]. Some important applications of MIONs in tissue regeneration research include tracking of transplanted cells; magnetically controlled release and delivery; magnetic regulation of proliferation, differentiation, and cell adhesion; and magnetothermal activation of ion channels and signal pathways and etc. [166] (Fig. 11).

Scientists are concentrating on the utilization of AuNPs to advance the detection and treatment of ocular disease. AuNPs, because of their anti-inflammatory and antiangiogenic characteristics, biocompatibility, low cytotoxicity, and passive nature are valuable for the detection and treatment of ocular disorders [170, 171]. The AuNPs' biocompatibility with ARPE-19 cells has been assessed by 2D and 3D confocal imaging. The findings revealed that AuNPs' biocompatibility and internalization are dependent on the size and shape of NPs [172].

For the development of a cell-based therapy approach for retinal degeneration, the human Wharton’s Jelly derived mesenchymal stem cells (WJ-MSCs) were loaded with AuNPs that led to low cellular deaths in the 10 days in comparison with the control. Besides, the histological assays revealed that the human WJ-MSCs transplantation in subretinal space postponed retinal degeneration without retinal tumorigenesis and systemic migration. Besides, confocal microscopy indicated the existence of human WJ-MSCs markers in photoreceptors, bipolar cells, and Müller cells [173]. With the progression in the semi-conductor method, the implantation of an electronic chip tool, which imitates cell behavior and treats retinopathies has become probable. Due to this, an investigation exhibited that micropatterned graphene oxide, after being combined with a retinal prosthesis, allowed the retinal cells adhesion. Additionally, GO-based nanomaterials offer increased safety, better tissue repair, and regeneration [174]. Also, a very elastic polyvinyl alcohol hydrogel with embedded nano-cellulose whiskers was produced as corneal implants and contact lenses [175]. Recently, it was shown that synthetic photoreceptors composed of AuNPs-decorated titania nanowire arrays have the ability for taking light and visual data processing in a photoreceptor degenerated retina [165]. Ranibizumab is an active humanized monoclonal antibody against VEGF A. A nano-formulation based on ranibizumab-conjugated Fe3O4/PEGylated polylactide-co-glycolide (PEG-PLGA) was produced for the neovascular AMD therapy. The in vitro assays exposed that Fe3O4-loaded PEG-PLGA polymer nanomaterial exhibited significant anti-angiogenic function. Furthermore, ranibizumab-conjugated PEG-PLGA showed an insignificant impact on human endothelial cell proliferation [88].

Bioactive and biocompatible nanomaterials could probably diminish problems related to corneal regeneration. Administration of peptide amphiphile (PA) nanofiber scaffolds modified with RGD (fibronectin) or YIGSR (laminin) sequences, into rabbit cornea have been used for the treatment of corneal wounds. The investigation following 3 and 7 weeks after injection with RGD exhibited considerable stromal keratinocytes migration and increased cornea regeneration [176]. Additionally, the cornea opacity was not affected by the treatment. Too, the incorporation of nano-silver into collagen hydrogels could generate collagen mimetic matrices with anti-microbial features [177]. Besides, rabbit corneal cells cultured on radially aligned nano-scaffolds exhibited a 1.2-fold enhancement in proliferation and gene expression than those grown on unaligned nano-scaffolds [178]. In comparison with microfiber scaffolds, nanofiber scaffolds result in less inflammatory responses, since they imitate mechanical characteristics of the natural cornea [179]. Though yet controversial, it was indicated that when the fiber size gets to the nano-range, the nanofiber diameter does not have any considerable impact on the activity and proliferation of corneal cells [180].

Nibourg et al. produced a nanogel based on self-assembled nanofiber composed of LMWG peptide to be applied as an extracellular medium for the growth of lens epithelial cells in a porcine ocular model. After eliminating the content of the lens, these nanogels were loaded into the porcine lens capsules, and then the filled lenses were removed from the porcine eye and were cultured for 3 weeks. The control sample was the lens filled with hyaluronan. In comparison with the control sample, the nanogels assisted the lens epithelial cells to preserve their normal morphology and had less expression of alpha-smooth muscle actin causing less capsular opacification. More improvement up to 10 times in capsular opacification was seen upon integrating various extracellular matrix (ECM)-derived peptides, such as YIGSR, IKVAV, DGEA, PHSRN, and RGDS [181]. To regenerate the corneal epithelium, gelatin nanofibers were used as a carrier of eyelid fat-derived stem cells. Stem cells cultured on aligned nanofibers had high cell viability and expressed the markers of corneal epithelial [182].

In the majority of nanowires that have been synthesized for retinal regeneration, AuNPs are loaded on the nanowires surface. For example, Tang et al. utilized titanium dioxide (TiO2) nanowire loaded by AuNPs. It has been demonstrated that TiO2 nanowire loaded AuNPs are able to effective inoculation of electrons in the implant and nanowires for the simulation of photoreceptors in the rat retina after photoillumination [165]. Some investigations have used stem cell-derived RPE-based treatments for AMD Therapy. Recently, a PLGA nano-scaffold has been applied for delivery of AMD-patient-derived iPSC RPE in porcine and rodent RPE injury AMD models [183]. The iPSC-RPE patches were safe, and after the degradation of PLGA scaffold, the AMD-iRPE patch was incorporated into Bruch’s membrane and was wholly functional. Moreover, many research have employed parylene-C implants and RPE for the treatment of vision loss in pre-clinical studies [184–186].

Julia Fernández-Pérez et al. acquired decellularized corneal ECM-based matrices using electrospinning to imitate the cornea fibrous structure. Fiber alignment and ECM combination impressed cell migration and morphology but the phenotype was not considerably affected. Keratocyte markers were enhanced in all scaffolds kinds compared with TCPS [187].

Tayebi et al. fabricated a biodegradable scaffold for cultivating corneal cells by integrating chitosan NPs (CSNPs) into polycaprolactone (PCL)/chitosan membranes. Numerous PCL/CSNP ratios were formulated in the presence of a fixed concentration of chitosan and the films were created using the solvent casting technique. The scaffold was not cytotoxic and upgraded the proliferation of HCECs as assessed through the MTT assay. In vitro assays such as H&E results, flow cytometry, cell counting, and SEM exhibited suitable attachment of HCECs to the scaffold which produced a compacted monolayer. The created scaffold appears to be appropriate for usage in the regeneration of corneal endothelial regarding biocompatibility and transparency [188].

## Advantages and disadvantages of the ocular nano-drug delivery systems (DDS)

The development of ocular nano-drug delivery systems (DDS) has made it feasible to overwhelm ocular-related barriers. Numerous hopeful carriers are accessible for ocular DDS, including liposomes, nanoemulsions, nanosuspensions, nanomicelles, and lipid and polymeric NPs [189]. Nanocarriers for ocular DDSs have many advantages. (1) They could penetrate the capillaries via the blood flows, enter the endothelial cell gap, and be taken in by pinocytosis to increase nanomaterial bioavailability. (2) Nanocarriers have a big surface area, which could embed hydrophobic substances, increase solubility and decrease the adverse effects of common solvents. (3) A nanocarrier could be modified via the targeting group to reorganize targeted drug delivery that may decrease the adverse effects, e. g. folic acid-modified NPs and MNPs [190]. (4) Nanocarriers extend the exclusion half-life of a substance, enhance efficient blood concentration-time, improve effectiveness, decrease use frequency, and diminish toxic effects. (5) Nanocarriers pass across the body obstacles that restrict the substance effects. (6) Nanocarriers have better interactions with drugs and conjunctival and corneal epithelium and increase the effectiveness of drug bioavailability and delivery [189, 191–193].

The main benefits of MNPs are that the NPs could be seen using MRI, and the drug-loaded NPs could be maintained in place using a magnetic field. One of the disadvantages of MNPs in drug delivery is their inability to concentrate in a three-dimensional space, because, the use of an external magnetic field organizes the MNPs into a two-dimensional space. Furthermore, it is challenging to retain the MNPs in the aimed tissue when the magnetic field is deleted from the outside. Another problem is associated with the time exposure to the magnetic field. The patients could not be continuously subjected to an external magnetic field, thus the therapeutic efficiency is restricted to the intensity, frequency, and exposure time of the magnetic field [194]. Polymeric nanomaterials have been utilized as the key element in different DDSs, probably owing to their outstanding features that comprise great water absorption ability, stability in an aqueous medium, brilliant biocompatibility, and similarity to alive tissues [185, 190, 195, 196].

Nevertheless, polymeric nanomaterials could influence vision following intraocular administration and be quickly removed from the blood flow. The success of SLNs depends on the development of dosage forms that have the capability to improve the therapeutic effects of the drugs via increasing their concentration, especially in the targeted tissues. Drugs could be combined in SLNs that cause propose a different model in drug delivery that can be used for drug targeting. The therapeutic load of numerous drug classes could be increased in particular organs by associating with SLNs. As well as, SLNs face many problems that comprise fast clearance, low stability, and non-specific uptake via the mononuclear phagocytic system [197]. The above restrictions could be nullified by combining diverse ligands to the SLNs surface that can assist to enhance the circulation time and targeted delivery of the drug to the particular site. The targeting features to a certain location could be increased by choosing surface biomarkers [198, 199].

Natural polysaccharides are fascinating choices for ophthalmic medicines, due to they are cheap, easily accessible, nontoxic, possibly biodegradable, commonly biocompatible, and usually compliant with chemical modifications [200–205]. Certainly, the chemical alteration has resulted in derivatives with better features in terms of enhanced residence time in the eye, and drug solubilization [206–210]. Some investigations concentrated on the polysaccharide-based nanocarriers' safety for the ocular sections; nevertheless, information on long-term surveillance and nanoformulation is yet absent [211–217]. Besides, the biodegradation of polysaccharide-based nanocarriers in the ocular system is not satisfying yet, even with the truth that polysaccharides simply degrade in vivo [218, 219].

In conclusion, at the nanoscale, several questions remain indistinct, for instance, how and where the polysaccharide-based nanocarrier is metabolized, decomposed, and defaecated; what metabolites are generated by their decomposition; and what is the probable influences on the physiological eye function, especially for hybrid nanomaterials. Evidence obtained has explained the biosafety and biocompatibility of polysaccharide-based nanocarriers based on material design, but there isn’t a good performance in actual situations. Regarding the impact of controlled-drug release in space and time assessment, some investigations on polysaccharide-based nanocarriers, particularly in physiological environments, keep on debatable owing to their differing research procedures and animal models [220, 221].

## Biosafety profiles and toxicity of nano-based materials for ocular drug delivery applications

While a considerable volume of information is accessible on the characterization, formulation, ophthalmic drug delivery, and nanomaterials targeting, data on the toxicity and safety of the above systems and materials are low. Toxicity and safety are critical subjects that are always of concern before to approval of ocular products for clinical studies [222]. Various investigations have recently been performed in this area. The combination of NLC and a thermoresponsive gel was suggested for the ocular injection of ibuprofen as an anti-inflammatory drug. This nanoformulation showed good biosafety and stability and extended ibuprofen release profile for ophthalmic delivery [223]. In another study, the efficiency of an intravitreal administration of liposomes-encapsulated infliximab in autoimmune uveoretinitis rat models was examined, and described that liposomes extend the drug stability in the vitreous and also showed acceptable biosafety and great therapeutic capacity in EAU [224].

Moreover, Tan et al exhibited that chitosan-coated liposomes had greater bioavailability and penetrability (3.9 and 2 times, respectively) compared to uncoated liposomes comprising a timolol maleate solution only. Besides, chitosan-modified liposomes and unmodified liposomes unceasingly release drugs in the eye tissues for 4 and 2 hours, respectively, which has a greater impact on decreasing IOP [225]. Compared to other nanomaterials, lipid-based nanocarriers such as liposomes and nanoemulsions were revealed to be safer and more biocompatible to interact with biomembranes and exhibited their existence in the market [3]. Cequa® has been examined for safety and effectiveness in treating dry eye syndrome. Phase III clinical trials with Cequa® were performed on 744 patients and also, the study design contained two 12 weeks of vehicle-controlled and randomized trials.

The findings showed a considerable enhancement in the Schirmer score from baseline with Cequa® relative to vehicle in tear generation with two-dose in a day. Furthermore, adverse effects were seen by more than 5% of patients [3]. Eudragit RL 100-based tacrolimus-loaded NP, was attained for local ocular use. The in vivo safety study exhibited no eye irritation via histopathological and ophthalmological analysis. This study reported the slow-release of tacrolimus from the particles and improved permeation of tacrolimus-loaded NPs to the eye compared with the solution drug [226].

RX-10045 is a dispersion of aqueous micelle of resolvin E1 prodrug. A Phase II clinical trial was done to evaluate the safety and effectiveness of RX-10045 in comparison with placebo for treating eye inflammation and pain after cataract surgery. Both RX-10045 formulations were not meaningfully superior to the placebo group in attaining the initial endpoint of clearance of anterior inflammation 8 days after cataract surgery [227].

MS Samimi et al, evaluated the irritation possibility and toxicity of fluconazole nanoemulsion in-situ gel formulation on retinal cells using HET-CAM, MTT, and Draize analyzes. The viability test on the retinal cells showed that fluconazole in-situ gel formulation was nontoxic and could be utilized in a safe mode in the ocular tissue at the 0.1% and 0.5% concentrations. Draize and HET-CAM assays exposed that fluconazole optimized formulation caused no irritation and was regarded as well-tolerated and non-irritant for ophthalmic use [228]. Similar efficacy and safety were seen in an assessment of a topical nanomicellar immunosuppressant formulation, everolimus. In this study, everolimus-loaded positively charged Soluplus was utilized to enhance penetrability via eye epithelia with no or minimal irritation leading to increased eye bioavailability for treatment of uveitis [229].

In conclusion, with regard to the eye sensitivity and the nanocarriers' toxicity, the safety of nanocarriers before using by patients is of big concern. The nanocarrier's appropriateness regarding biodegradability, sustained or burst release, and patient convenience for diverse clinical requirements in the anterior segment still should be wholly investigated. As well, consideration should be given to nanocarrier biological interactions and surface chemistry as they could help to better comprehension of the biosafety profile of nanocarriers [230].

## FDA approved and under clinical trial nanomedicine for ocular diseases

Significant investigations have been performed on nanomedicines for relieving anterior and posterior ocular diseases. They have shown positive results in clinical trials [231]. Restasis (cyclosporine A nanoemulsion), as the first commercial product, was developed to treat dry eye syndrome and Durezol (difluprednate nanoemulsion), is approved to treat eye inflammation [232]. Other commercial ocular nanostructured products utilized to treat dry eye diseases include Cequa® [231], and Cyclokat® [233], which are both nanoemulsion formulations of Cyclosporin A; and also, Lacrisek® (liposomal vitamin E and vitamin A-palmitate), and Artelac Rebalance® (Lubricant). Furthermore, another FDA-approved drug named Ikervis® (cationic nanoemulsion) has been designed for acute keratitis therapy in dry eye syndrome [231]. Visudyne, as an ophthalmic nanomedicine, has been FDA approved for treating choroidal neovascularization, ocular histoplasmosis syndrome, and pathological myopia. Macugen®, as a FDA-approved nanodrug, is a PEG anti-VEGF aptamer, administered by intravitreal injection for wet AMD therapy [234]. InSite Vision has formulated a novel drug delivery system named Durasite® as the basis for a wide range of ophthalmic drugs. A DuraSite formulation comprising Besifloxacin was FDA approved for the treatment of bacterial pink eye [5]. Besides, several ophthalmic nanomedicines, have been FDA approved for treating macular edema including Ozurdex (dexamethasone biodegradable implant) [235] and Iluvien (Fluocinolone acetonide) as a nonbiodegradable implant [236], as well as, Triesence (Triamcinolone acetonide suspension) [194, 237] and Kenalog (Triamcinolone acetonide suspension) [194, 237] that are administrated as microparticles. Also, Retisert (Fluocinolone acetonide nonbiodegradable implant) [238] and Trivaris (Triamcinolone acetonide suspension) [194, 237], administered by intravitreal injection, were approved for the treatment of uveitis. AzaSite (Azithromycin Ophthalmic 1% Solution), administrated as an eye drop, has been approved for bacterial conjunctivitis therapy. Numerous ocular nanoformulations such as TLC399 (ProDex) that are used for macular edema therapy, are currently in phase II clinical trial. Moreover, latanoprost-coated liposome (POLAT-001) has done phase II clinical trials for ophthalmic hypertension and initial treatment of open angle glaucoma [239]. SYSTANE®, one recent ophthalmic nanoemulsion, has done phase IV clinical trial for the treatment of dry eye syndrome [193]. Several clinical trials are ongoing, such as a study for evaluation of the efficacy and safety study of ENV 515 for the treatment of ocular hypertension (NCT02371746) and glaucoma. AR-13503 (NCT03835884) and AR-1105 (NCT03739593) were designed as intravitreal implants for treating AMD and diabetic macular edema [3, 240]. Several other under clinical trial ophthalmic nanomedicines, include Taxol (Paclitaxel albumin-stabilized nanoparticle formulation), which has done phase II clinical trial for treating intraocular melanoma [241]; GB-102 (Sunitinib malate), in phase I clinical trial for AMD therapy [242]; KPI-121 (1% and 0.25% loteprednol etabonate), is currently in phase III clinical trial for the treatment of ocular infection, irritation and inflammation [243] and OCS-01 (dexamethasone cylcodextrin) has done phase II clinical trial for treating eye inflammation and pain [244] (Table 2). By enhancing the nano-based material numbers on the market or in the clinical studies, it seems that the development of nano-based technologies in ocular disease treatment is a hopeful strategy, though further studies and research are needed for the delivery of nanostructure to the eye [193].

**Table 2:** FDA approved and under clinical trial nanomedicine for ocular diseases

Trade name	Drug/Bioactives		Target tissue	Target indication (Use)	Delivery system	Route	FDA Approval Status	Refs.
Durezol®		Difluprednate Ophthalmic Emulsion 0.05%	Mainly anterior segment of the eye, anterior chamber, cornea conjunctiva	Anterior uveitis	Nanoemulsion	Eye drop	Approved	[274]
Restasis®		Cyclosporine ophthalmic emulsion 0.05%	Cornea and Tear film	Dry eye	Nanoemulsion	Oral, intravenous (IV), eye drop	Approved	[275]
Ikervis®	Ciclosporin ophthalmic emulsion 0.1%		Cornea and Tear film	Dry eye	Nanoemulsion	Eye drop	Approved	[276]
Cequa®	Cyclosporine ophthalmic solution 0.09%		Cornea and Tear film	Dry eye	Micelle	Eye drop	Approved	[277]
Cyclokat®	Cationic emulsion 0.1%		Cornea and Tear film	Dry eye	Cationic nanoemulsion	Eye drop	Approved	[278]
Lacrisek®	Vitamin A palmitate, vitamin E		Cornea and Tear film	Dry eye	Liposomal spray	Eye drop	Approved	[279]
Artelac Rebalance®	Lubricant		Cornea and Tear film	Dry eye	Liposomal eyedrops	Eye drop	Approved	[280]
Ozurdex	Dexamethasone biodegradable implant		Vitreous, Retina, Choroid	Macular edema, Non-infectious uveitis	Implant	Intravitreal injection	Approved	[235]
Iluvien	Fluocinolone acetonide nonbiodegradable implant		Vitreous, Retina, Choroid	Diabetic macular edema	Implant	Intravitreal injection	Approved	[236]
Visudyne	Verteporfin		Retina, Choroid	Wet AMD, Choroidal neovascularization, Central Serous Chorioretinopathy	Liposomal injection	Intravenous	Approved	[281]
Macugen®	Pegaptanib		Retina Choroid	Wet AMD	Liposome	Intravitreal injection	Approved	[282]
Triesence	Triamcinolone acetonide suspension		Vitreous, Retina, Choroid	Macular edema	Microparticle	Intravitreal injection	Approved	[194, 237]
AzaSite ®	Azithromycin Ophthalmic 1% Solution		Conjunctiva, cornea	Antimicrobial	Micelle	Eye drop	Approved	[283]
Trivaris	Triamcinolone acetonide suspension		Vitreous, Retina, Choroid	Uveitis	Microparticle	Intravitreal injection	Approved	[194, 237]
Kenalog	Triamcinolone acetonide suspension		Vitreous, Retina, Choroid	Macular edema	Microparticle	Intravitreal injection, Suprachoroidal injection	Approved	[194, 237]
Retisert	Fluocinolone acetonide nonbiodegradable implant		Vitreous, Retina, Choroid	Uveitis	Implant	Intravitreal injection	Approved	[238]
TLC399	ProDex		Vitreous, Retina, Choroid	Macular edema	Pro Dex	Intravitreal injection	Phase II	[239]
POLAT-001	latanoprost-coated liposome		Anterior segment	Glaucoma	Liposome	Subconjunctival injection	Phase II	[284]
SYSTANE®	Propylene glycol-based nanoemulsion		Cornea and Tear film	Dry eye	Nanoemulsion	Eye drop	Phase IV	[285]
ENV 515	Travoprost extended release (XR)		Anterior segment	Glaucoma	Nanoparticles	Intracameral Implant	Phase II	[286]
AR-13503	AR-13503 implant alone and in combination with aflibercept		Retina, Choroid	Neovascular AMD and diabetic macular edema	Intravitreal implants	Intravitreal injection	Phase I	[3, 240]
AR-1105	Dexamethasone intravitreal implant		Retina, Choroid	AMD and diabetic macular edema	Intravitreal implants	Intravitreal injection	Phase II	[3]
Taxol	Paclitaxel albumin-stabilized nanoparticle formulation		Retina, Choroid	Intraocular melanoma	Nanoparticles	Intravenous injection	Phase II	[241]
GB-102	Sunitinib malate		Retina, Choroid	AMD	Nanoparticles	Intravitreal injection	Phase I	[242]
KPI-121	1 and 0.25% loteprednol etabonate		Anterior segment	Conjunctiva, cornea, tear film	Mucus penetrating particles	Eye drop	Phase III	[243]
OCS-01	Dexamethasone Cylcodextrin Nanoparticle Ophthalmic Suspension 1.5% mg/ml		Anterior segment, Retina	Control of Inflammation, Diabetic Macular Edema	Nanoparticle	Eye drop	Phase II	[244]

## Conclusions, challenges and perspectives

Due to the anatomical barriers and physiological conditions of the eye, effective delivery of ocular medicine is a big challenge to pharmacologists and researchers [5]. The topical administration route is the most widely applied noninvasive method of drug delivery for treating anterior eye segment diseases. Conventional ophthalmic formulations such as eye drops account for 90% of the market. The reason might be ascribed to patient compliance and ease of administration [245, 246]. However, the ocular drug bioavailability with topical administration of eye drop is very low, and less than 5% of the topically used drug reaches deeper eye tissues [247]. As well, it is challenging to reach therapeutic drug concentration in the posterior eye segment after topical administration of eye drops. The drug could be delivered to the posterior eye segment by different administration modes such as systemic administration, periocular injections, and intravitreal injections. Intravitreal injection is the most common and frequently used drug administration route for the treatment of posterior segment eye diseases. Although, the repeated eye puncture with intravitreal injections causes several adverse effects such as hemorrhage, endophthalmitis, and retinal detachment [248]. Drug delivery with the periocular administration route is developed as an alternative method of drug delivery to the posterior eye segments. Though this drug administration route is relatively less invasive, easy, and patient compliant, drug penetration is compromised by ocular dynamic and static barriers [40, 249, 250].

Nanotechnology has used different nanocarriers to develop potential ophthalmic drug delivery systems to be efficient at overcoming the limitations associated with conventional ocular dosage forms. Many nanocarriers, including magnetic nanoparticles, polymeric nanoparticles, liposomes, nanomicelles, etc. have been investigated for improved penetration and efficient targeted drug delivery to various ocular sites [3, 251]. Among the various types of nanomaterials used for ocular drug delivery, we found that polymeric nanomaterials have been the focus of significant attention throughout the last years as evidenced by an unprecedented enhancement in the number of research papers concentrating on these materials. The reasons for the popularity of polymeric nanomaterials include their mucosal adhesion property that increases the drug's shelf life in the eye and prevents rapid drug clearance. Also, the stimulus-responsive properties that permit them to release an active ingredient after a change in conditions, and the biodegradability of polymers have made them attractive materials as ocular nanomedicine [115].

The considerable advantages that are commonly present in most existing ocular nanomedicines include great stability and biodegradability, low cytotoxicity, high biocompatibility and bioavailability, high surface area and pore volume, controlled release, and their capability to penetrate across complex ocular barriers, particularly BRB and the corneal-retinal barrier, with minimal unwanted ocular/systematic adverse effects [193, 252]. However, common side effects commonly seen in ocular nanomedicines include blurry vision, sensitivity to light, eye irritation, eye redness, pain, corneal edema, raised intraocular pressure, infection, conjunctivitis, eye discharge, and headache [250, 253].

Despite the significant advantages of nanomedicine over routine ocular administration routes, all methods are yet restricted to preclinical studies with numerous challenges that require to be overcome, for example, late-phase clinical trials and large-scale manufacturing to allow scholars to attain robust evidence and findings. Besides, future advances in a standard nanoscale clinical drug delivery vehicle need to concentrate on the heterogeneous appearances of the disease, such as the pathogenesis and etiology. The effects of surface charge, particle size, aggregation, and composition on the pharmaco-toxic profiles and pharmacokinetics should be determined [251]. Some nanomaterials are utilized to fabricate nanosystems, but their toxicity has not been entirely clear to the eye, particularly for those materials which require frequent doses. There are some other challenges that need to be addressed including: among many studies of ophthalmic disease treatment by nanotechnology, fewer in vivo studies have been done and more studies are concentrated on in vitro research. Additional attempts must be made on animal models particularly the eye cancers model should be developed in the future. While the rabbit is the most frequently used animal due to the comparable size of the human eye, surface sensitivity and mucus production are higher in rabbit eyes, but the blinking number and tear production in rabbits is less compared to humans [254]. These differences can make the nanosystem's effect unauthentic to human beings [34, 255, 256].

The use of active targeting ligands to incorporate penetration enhancers into composite systems or modifying nano-formulations might be an efficient approach for ocular nano-carrier DDSs for drug delivery to the posterior eye segment, but the studies about the uptake of targeted nano-carriers in the treating posterior segment ophthalmic disorders are rare. Investigations are required to fill these gaps and prevail the complications associated with nanocarriers functionalized with many ligands of tissues and cells of the posterior eye segment as vehicles [251].

It appears that some colloidal nanocarriers and FDA-approved nanomaterials have further capacity in use. Besides delivery systems, noninvasive delivery methods will be highlighted in the future for ophthalmic disorders in the anterior and posterior segments. Ultimately, all-in-one systems that may integrate diagnostic and therapeutic functions might be presented to facilitate visual follow-up throughout eye disease therapy. Table 3 limits some features of the reported nano-based materials for the diagnosis and treatment of ocular disease.

**Table 3:** Summary of the reported nano-based materials for diagnosis and treatment of ocular diseases

Material	Target	Specific feature	Advantages	Refs.
Ranibizumab-loaded NPs (S-PEG-ICG-RGD-RBZ)	Choroidal neovascularization	Antibody-NPs conjugates	Low cytotoxicity and genotoxicity, no apoptosis	[56]
MSIO nanofluid, PEGylated Fe2O4	Glaucoma Therapy	Magnetic core and polyethylene glycol (PEG) surface coating	High biocompatibility, high cellular uptake, low cytotoxicity	[86]
Avastin–Fe3O4 nanocomposites	AMD therapy	Antibody-NPs conjugates	Long-term release of Avastin	[87]
Ranibizumab conjugated iron oxide (Fe3O4)/PEGylated PLGA	AMD therapy	Antibody-conjugated nanoparticles	More efficient drug delivery and better inhibition of tube formation	[88]
valproic acid and guanabenz -loaded MNPs	Barded-Biedl syndrome (BBS)	Magnetically assisted delivery system	Non-invasive and needle-free technology	[90]
Ranibizumab/PEG-conjugated AuNPs	Angiogenesis-associated disorders such as AMD	Antibody-conjugated nanoparticles	Long half-life of Mab, protection of Mab from the high protease	[95]
Resveratrol-coated gold NPs	diabetic retinopathy	–	No toxicity	[97]
Ranibizumab -conjugated MNPs	Eye disorders	Antibody-conjugated nanoparticles	No cytotoxicity	[99]
Nanodiscs and gold nanorods	Early detection of diabetic retinopathy	Urine based colorimetric test paper linked with a smartphone	High specificity and sensitivity	[99]
ST/FA-b-PEG-AuNPs@G	Diabetic retinopathy therapy	Site specific drug delivery	Effective drug delivery and controlled drug release	[100]
HA-gold NPs	Ocular neovascularization-related diseases	Particular receptor interaction	Increased distribution and stability	[101]
Pilocarpine-encapsulated MSNs gelatin-covered	Reduction of IOP	Gelatin-covered	Progressive and continuous drug leakage	[108]
Silica-coated Au nanorods	Prevention of posterior capsule opacification (PCO)	Spatial controllability of photothermal effect	Prevention of disordered LECs fibrosis formation, elimination of residual lens epithelial cells around Nano-IOLs	[110]
CeCl3@mSiO2 NPs	Treatment of diabetic cataract	–	Antioxidant effect	[111]
Reverse thermoresponsive polymer (RTP)	AMD therapy	Thermoresponsivity	Nontoxic, Sustained-release intraocular drug delivery vehicle, slowly releases anti-VEGF agents, in vitro and in vivo biocompatibility	[124]
Axitinib-loaded MPEG-PCL micelles	Treatment of ophthalmic neovascular disorders	–	Great cell biocompatibility, low toxicity	[132]
Timolol maleate (TML)-loaded polymeric NPs	Glaucoma therapy	–	Significant bioadhesive ability, sustained drug release, good biocompatibility	[147]
Glycyrrhizin-based self-assembled nanomicelles	Treatment of inflammation-, oxidative stress- and bacteria-related ocular diseases	–	Improved in vitro release and antioxidant activity	[134]
Atorvastatin (ATS)—SLNs	AMD therapy	–	Great bioavailability, good ocular safety and stability, extended retention time	[133]
Bimatoprost (BIM) NPs-loaded pH-sensitive in-situ gel	Glaucoma therapy	pH-sensitivity	Improved drug release, well-tolerated, nonirritant	[130]
Amphotericin B (AmB)-loaded PEGylated-NLC	Treatment of ocular disease	PEGylation	No toxicity, improved drug loading	[131]
Hyaluronan-cholesterol nanogels (NHs)	Treatment of anterior/posterior eye segment disorders	–	Enhanced the ocular bioavailability, increased permeation of loaded drugs	[160]
Pullulan–dexamethasone	Retinal disease treatment	–	Good safety, extended residence time and controlled-release	[162]

## Data Availability

Not applicable.

## References

[1] Nagaraj R, Bijukumar DR, Mathew B, Scott EA, Mathew MT (2019). A review on recent advancements in ophthalmology devices: currently in market and under clinical trials. J Drug Deliv Sci Technol.

[2] Balantrapu T. Latest global blindness & VI prevalence figures published in Lancet. 2018. www.iapb.org/news/latest-global-blindness-vi-prevalence-figures-published-lancet. Accessed 5 Jan 2017.

[3] Gorantla S, Rapalli VK, Waghule T, Singh PP, Dubey SK, Saha RN (2020). Nanocarriers for ocular drug delivery: current status and translational opportunity. RSC Adv.

[4] Schoenfeld ER, Greene JM, Wu SY, Leske MC (2001). Patterns of adherence to diabetes vision care guidelines: baseline findings from the Diabetic Retinopathy Awareness Program. Ophthalmology.

[5] Weng Y, Liu J, Jin S, Guo W, Liang X, Hu Z (2017). Nanotechnology-based strategies for treatment of ocular disease. Acta Pharm Sin B.

[6] Amirsaadat S, Jafari-Gharabaghlou D, Alijani S, Mousazadeh H, Dadashpour M, Zarghami N (2021). Metformin and Silibinin co-loaded PLGA-PEG nanoparticles for effective combination therapy against human breast cancer cells. J Drug Deliv Sci Technol.

[7] Adlravan E, Nejati K, Karimi MA, Mousazadeh H, Abbasi A, Dadashpour M (2021). Potential activity of free and PLGA/PEG nanoencapsulated nasturtium officinale extract in inducing cytotoxicity and apoptosis in human lung carcinoma A549 cells. J Drug Deliv Sci Technol.

[8] Mousazadeh H, Pilehvar-Soltanahmadi Y, Dadashpour M, Zarghami N (2021). Cyclodextrin based natural nanostructured carbohydrate polymers as effective non-viral siRNA delivery systems for cancer gene therapy. J Control Release.

[9] Bargahi N, Ghasemali S, Jahandar-Lashaki S, Nazari A (2022). Recent advances for cancer detection and treatment by microfluidic technology, review and update. Biol Proced Online.

[10] Ghasemali S, Farajnia S, Barzegar A, Rahmati-Yamchi M, Baghban R, Rahbarnia L (2021). New developments in anti-angiogenic therapy of cancer, review and update. Anticancer Agents Med Chem.

[11] Raghava S, Goel G, Kompella UB. Ophthalmic applications of nanotechnology. In: Tombran-Tink J, Barnstable CJ, editors. Ocular transporters in ophthalmic diseases and drug delivery. Humana Press; 2008. p. 415–35. 10.1007/978-1-59745-375-2_22. Print ISBN: 978-1-58829-958-1, Online ISBN: 978-1-59745-375-2.

[12] Amrite AC, Kompella UB. Nanoparticles for ocular drug delivery. Nanoparticle technology for drug delivery. CRC Press; 2006. p. 343-84. 10.1201/9780849374555.ch11

[13] Kompella UB, Amrite AC, Ravi RP, Durazo SA (2013). Nanomedicines for back of the eye drug delivery, gene delivery, and imaging. Prog Retin Eye Res.

[14] Ahmadkhani L, Mostafavi E, Ghasemali S, Baghban R, Pazoki-Toroudi H, Davaran S (2019). Development and characterization of a novel conductive polyaniline-g-polystyrene/Fe3O4 nanocomposite for the treatment of cancer. Artif Cells Nanomed Biotechnol.

[15] Tang Z, Fan X, Chen Y, Gu P (2022). Ocular nanomedicine. Adv Sci.

[16] Barani M, Sabir F, Rahdar A, Arshad R, Kyzas GZ (2020). Nanotreatment and nanodiagnosis of prostate cancer: recent updates. Nanomaterials.

[17] Barani M, Mukhtar M, Rahdar A, Sargazi G, Thysiadou A, Kyzas GZ (2021). Progress in the application of nanoparticles and graphene as drug carriers and on the diagnosis of brain infections. Molecules.

[18] Barani M, Nematollahi MH, Zaboli M, Mirzaei M, Torkzadeh-Mahani M, Pardakhty A (2019). In silico and in vitro study of magnetic niosomes for gene delivery: the effect of ergosterol and cholesterol. Mater Sci Eng C.

[19] Das SS, Bharadwaj P, Bilal M, Barani M, Rahdar A, Taboada P (2020). Stimuli-responsive polymeric nanocarriers for drug delivery, imaging, and theragnosis. Polymers.

[20] Davarpanah F, Yazdi AK, Barani M, Mirzaei M, Torkzadeh-Mahani M (2018). Magnetic delivery of antitumor carboplatin by using PEGylated-Niosomes. DARU J Pharm Sci.

[21] Ebrahimi AK, Barani M, Sheikhshoaie I (2018). Fabrication of a new superparamagnetic metal-organic framework with core-shell nanocomposite structures: characterization, biocompatibility, and drug release study. Mater Sci Eng C.

[22] Ghazy E, Rahdar A, Barani M, Kyzas GZ (2021). Nanomaterials for Parkinson disease: recent progress. J Mol Struct.

[23] Hajizadeh MR, Maleki H, Barani M, Fahmidehkar MA, Mahmoodi M, Torkzadeh-Mahani M (2019). In vitro cytotoxicity assay of D-limonene niosomes: an efficient nano-carrier for enhancing solubility of plant-extracted agents. Res Pharm Sci.

[24] Zahin N, Anwar R, Tewari D, Kabir M, Sajid A, Mathew B (2020). Nanoparticles and its biomedical applications in health and diseases: special focus on drug delivery. Environ Sci Pollut Res.

[25] Si X-Y, Merlin D, Xiao B (2016). Recent advances in orally administered cell-specific nanotherapeutics for inflammatory bowel disease. World J Gastroenterol.

[26] Bonilla L, Espina M, Severino P, Cano A, Ettcheto M, Camins A (2021). Lipid nanoparticles for the posterior eye segment. Pharmaceutics.

[27] Begines B, Ortiz T, Pérez-Aranda M, Martínez G, Merinero M, Argüelles-Arias F (2020). Polymeric nanoparticles for drug delivery: Recent developments and future prospects. Nanomaterials.

[28] Cunha-Vaz J, Bernardes R, Lobo C (2011). Blood-retinal barrier. Eur J Ophthalmol.

[29] Chong DY, Johnson MW, Huynh TH, Hall EF, Comer GM, Fish DN (2009). Vitreous penetration of orally administered famciclovir. Am J Ophthalmol.

[30] Srinivas A, Azad RV, Sharma YR, Kumar A, Satpathy G, Velpandian T (2009). Evaluation of vitreous levels of gatifloxacin after systemic administration in inflamed and non-inflamed eyes. Acta Ophthalmol.

[31] Kim H, Robinson MR, Lizak MJ, Tansey G, Lutz RJ, Yuan P (2004). Controlled drug release from an ocular implant: an evaluation using dynamic three-dimensional magnetic resonance imaging. Invest Ophthalmol Vis Sci.

[32] Janoria KG, Gunda S, Boddu SH, Mitra AK (2007). Novel approaches to retinal drug delivery. Expert Opin Drug Deliv.

[33] Shah SS, Denham LV, Elison JR, Bhattacharjee PS, Clement C, Huq T (2010). Drug delivery to the posterior segment of the eye for pharmacologic therapy. Expert Rev Ophthalmol.

[34] Marmor MF, Negi A, Maurice DM (1985). Kinetics of macromolecules injected into the subretinal space. Exp Eye Res.

[35] SomsanguanAusayakhun M, Yuvaves P (2005). Treatment of cytomegalovirus retinitis in AIDS patients with intravitreal ganciclovir. J Med Assoc Thai.

[36] Ranta V-P, Urtti A (2006). Transscleral drug delivery to the posterior eye: prospects of pharmacokinetic modeling. Adv Drug Deliv Rev.

[37] Ambati J, Adamis AP (2002). Transscleral drug delivery to the retina and choroid. Prog Retin Eye Res.

[38] Geroski DH, Edelhauser HF (2001). Transscleral drug delivery for posterior segment disease. Adv Drug Deliv Rev.

[39] Raghava S, Hammond M, Kompella UB (2004). Periocular routes for retinal drug delivery. Expert Opin Drug Deliv.

[40] Kim SH, Lutz RJ, Wang NS, Robinson MR (2007). Transport barriers in transscleral drug delivery for retinal diseases. Ophthalmic Res.

[41] Ranta V-P, Mannermaa E, Lummepuro K, Subrizi A, Laukkanen A, Antopolsky M (2010). Barrier analysis of periocular drug delivery to the posterior segment. J Control Release.

[42] Thrimawithana TR, Young S, Bunt CR, Green C, Alany RG (2011). Drug delivery to the posterior segment of the eye. Drug Discov Today.

[43] Gaudana R, Ananthula HK, Parenky A, Mitra AK (2010). Ocular drug delivery. AAPS J.

[44] Patel SR, Lin AS, Edelhauser HF, Prausnitz MR (2011). Suprachoroidal drug delivery to the back of the eye using hollow microneedles. Pharm Res.

[45] Einmahl S, Savoldelli M, Dhermies FO, Tabatabay C, Gurny R, Behar-Cohen F (2002). Evaluation of a novel biomaterial in the suprachoroidal space of the rabbit eye. Invest Ophthalmol Vis Sci.

[46] Olsen TW, Feng X, Wabner K, Conston SR, Sierra DH, Folden DV (2006). Cannulation of the suprachoroidal space: a novel drug delivery methodology to the posterior segment. Am J Ophthalmol.

[47] Liu S, Liu W, Ma Y, Liu K, Wang M (2012). Suprachoroidal injection of ketorolac tromethamine does not cause retinal damage. Neural Regen Res.

[48] Ghate D, Brooks W, McCarey BE, Edelhauser HF (2007). Pharmacokinetics of intraocular drug delivery by periocular injections using ocular fluorophotometry. Ophthalmol Vis Sci.

[49] Singh SR, Dogra M, Singh R, Dogra MR (2019). Accidental globe perforation during posterior sub-tenon's injection of triamcinolone acetonide. Ophthalmic Surg Lasers Imaging Retina.

[50] Thorne JE, Sugar EA, Holbrook JT, Burke AE, Altaweel MM, Vitale AT (2019). Periocular triamcinolone vs. intravitreal triamcinolone vs. intravitreal dexamethasone implant for the treatment of uveitic macular edema: the PeriOcular vs. INTravitreal corticosteroids for uveitic macular edema (POINT) trial. Ophthalmology.

[51] Sen HN, Vitale S, Gangaputra SS, Nussenblatt RB, Liesegang TL, Levy-Clarke GA (2014). Periocular corticosteroid injections in uveitis: effects and complications. Ophthalmology.

[52] Lafranco Dafflon M, Tran VT, Guex-Crosier Y, Herbort CP (1999). Posterior sub-Tenon’s steroid injections for the treatment of posterior ocular inflammation: indications, efficacy and side effects. Graefes Arch Clin Exp Ophthalmol.

[53] Ghazy E, Kumar A, Barani M, Kaur I, Rahdar A, Behl T (2021). Scrutinizing the therapeutic and diagnostic potential of nanotechnology in thyroid cancer: edifying drug targeting by nano-oncotherapeutics. J Drug Deliv Sci Technol.

[54] Honda M, Asai T, Oku N, Araki Y, Tanaka M, Ebihara N (2013). Liposomes and nanotechnology in drug development: focus on ocular targets. Int J Nanomedicine.

[55] Bulbake U, Doppalapudi S, Kommineni N, Khan W (2017). Liposomal formulations in clinical use: an updated review. Pharmaceutics.

[56] Cai W, Chen Q, Shen T, Yang Q, Hu W, Zhao P (2020). Intravenous anti-VEGF agents with RGD peptide-targeted core cross-linked star (CCS) polymers modified with indocyanine green for imaging and treatment of laser-induced choroidal neovascularization. Biomater Sci.

[57] Nguyen VP, Qian W, Li Y, Liu B, Aaberg M, Henry J (2021). Chain-like gold nanoparticle clusters for multimodal photoacoustic microscopy and optical coherence tomography enhanced molecular imaging. Nat Commun.

[58] Golabchi K, Soleimani-Jelodar R, Aghadoost N, Momeni F, Moridikia A, Nahand JS (2018). MicroRNAs in retinoblastoma: potential diagnostic and therapeutic biomarkers. J Cell Physiol.

[59] Chen X-J, Zhang X-Q, Liu Q, Zhang J, Zhou G (2018). Nanotechnology: a promising method for oral cancer detection and diagnosis. J Nanobiotechnology.

[60] Mukhtar M, Bilal M, Rahdar A, Barani M, Arshad R, Behl T (2020). Nanomaterials for diagnosis and treatment of brain cancer: Recent updates. Chemosensors.

[61] Nikazar S, Barani M, Rahdar A, Zoghi M, Kyzas GZ (2020). Photo-and magnetothermally responsive nanomaterials for therapy, controlled drug delivery and imaging applications. ChemistrySelect.

[62] Rahdar A, Taboada P, Hajinezhad MR, Barani M, Beyzaei H (2019). Effect of tocopherol on the properties of Pluronic F127 microemulsions: physico-chemical characterization and in vivo toxicity. J Mol Liq.

[63] Sabir F, Barani M, Rahdar A, Bilal M, Nadeem M (2021). How to face skin cancer with nanomaterials: a review. Biointerface Res Appl Chem.

[64] Zhang Y, Li M, Gao X, Chen Y, Liu T (2019). Nanotechnology in cancer diagnosis: progress, challenges and opportunities. J Hematol Oncol.

[65] Moradi S, Mokhtari-Dizaji M, Ghassemi F, Sheibani S, Asadi AF (2020). Increasing the efficiency of the retinoblastoma brachytherapy protocol with ultrasonic hyperthermia and gold nanoparticles: a rabbit model. Int J Radiat Biol.

[66] Nguyen VP, Li Y, Qian W, Liu B, Tian C, Zhang W (2019). Contrast agent enhanced multimodal photoacoustic microscopy and optical coherence tomography for imaging of rabbit choroidal and retinal vessels in vivo. Sci Rep.

[67] Lapierre-Landry M, Gordon AY, Penn JS, Skala MC (2017). In vivo photothermal optical coherence tomography of endogenous and exogenous contrast agents in the eye. Sci Rep.

[68] Tzameret A, Ketter-Katz H, Edelshtain V, Sher I, Corem-Salkmon E, Levy I (2019). In vivo MRI assessment of bioactive magnetic iron oxide/human serum albumin nanoparticle delivery into the posterior segment of the eye in a rat model of retinal degeneration. J Nanobiotechnology.

[69] Jaidev L, Chellappan DR, Bhavsar DV, Ranganathan R, Sivanantham B, Subramanian A (2017). Multi-functional nanoparticles as theranostic agents for the treatment & imaging of pancreatic cancer. Acta Biomater.

[70] Arshad R, Barani M, Rahdar A, Sargazi S, Cucchiarini M, Pandey S (2021). Multi-functionalized nanomaterials and nanoparticles for diagnosis and treatment of retinoblastoma. Biosensors.

[71] Salmani Javan E, Lotfi F, Jafari-Gharabaghlou D, Mousazadeh H, Dadashpour M, Zarghami N (2022). Development of a magnetic nanostructure for co-delivery of metformin and silibinin on growth of lung cancer cells: Possible action through leptin gene and its receptor regulation. Asian Pac J Cancer Prev.

[72] Ito A, Shinkai M, Honda H, Kobayashi T (2005). Medical application of functionalized magnetic nanoparticles. J Biosci Bioeng.

[73] Reyes-Ortega F, Delgado ÁV, Iglesias GR (2021). Modulation of the magnetic hyperthermia response using different superparamagnetic iron oxide nanoparticle morphologies. Nanomaterials.

[74] Tan M, Reyes-Ortega F, Schneider-Futschik EK (2020). Successes and challenges: inhaled treatment approaches using magnetic nanoparticles in cystic fibrosis. Magnetochemistry.

[75] Avasthi A, Caro C, Pozo‑Torres E, Leal MP, García‑Martín ML. Magnetic nanoparticles as MRI contrast agents. In: Surface-modified Nanobiomaterials for Electrochemical and Biomedicine Applications 2020, 49–91. 10.1007/978-3-030-55502-3_3

[76] Shabatina TI, Vernaya OI, Shabatin VP, Melnikov MY (2020). Magnetic nanoparticles for biomedical purposes: modern trends and prospects. Magnetochemistry.

[77] Nejati K, Dadashpour M, Gharibi T, Mellatyar H, Akbarzadeh A (2021). Biomedical applications of functionalized gold nanoparticles: a review. J Clust Sci.

[78] Malhotra N, Lee J-S, Liman RAD, Ruallo JMS, Villaflores OB, Ger T-R (2020). Potential toxicity of iron oxide magnetic nanoparticles: A review. Molecules.

[79] Arruebo M, Fernández-Pacheco R, Ibarra MR, Santamaría J (2007). Magnetic nanoparticles for drug delivery. Nano Today.

[80] Xie J, Chen K, Huang J, Lee S, Wang J, Gao J (2010). PET/NIRF/MRI triple functional iron oxide nanoparticles. Biomaterials.

[81] Baghban R, Afarid M, Soleymani J, Rahimi M (2021). Were magnetic materials useful in cancer therapy?. Biomed Pharmacother.

[82] Pankhurst QA, Connolly J, Jones SK, Dobson J (2003). Applications of magnetic nanoparticles in biomedicine. J Phys D.

[83] Amsalem Y, Mardor Y, Feinberg MS, Landa N, Miller L, Daniels D (2007). Iron-oxide labeling and outcome of transplanted mesenchymal stem cells in the infarcted myocardium. Circulation.

[84] Yanai A, Häfeli UO, Metcalfe AL, Soema P, Addo L, Gregory-Evans CY (2012). Focused magnetic stem cell targeting to the retina using superparamagnetic iron oxide nanoparticles. Cell Transplant.

[85] Giannaccini M, Pedicini L, Di Leo N, Giannini M, Calatayud M, Goya G, et al. Nanoparticles as drug carrier for the posterior chamber of the eye. In: BioNanoMed 2015 - Abstract book; 2015.

[86] Bae S, Jeoung JW, Jeun M, Jang J-T, Park JH, Kim YJ (2016). Magnetically softened iron oxide (MSIO) nanofluid and its application to thermally-induced heat shock proteins for ocular neuroprotection. Biomaterials.

[87] Zargarzadeh M, MadaahHosseini HR, Delavari H, Irajirad R, Aghaie E (2018). Synthesis of magnetite (Fe3O4)—avastin nanocomposite as a potential drug for AMD treatment. Micro Nano Lett.

[88] Yan J, Peng X, Cai Y, Cong W (2018). Development of facile drug delivery platform of ranibizumab fabricated PLGA-PEGylated magnetic nanoparticles for age-related macular degeneration therapy. J Photochem Photobiol B Biol.

[89] Demirci H, Slimani N, Pawar M, Kumon RE, Vaishnava P, Besirli CG (2019). Magnetic hyperthermia in Y79 retinoblastoma and ARPE-19 retinal epithelial cells: tumor selective apoptotic activity of iron oxide nanoparticle. Transl Vis Sci Technol.

[90] Bassetto M, Ajoy D, Poulhes F, Obringer C, Walter A, Messadeq N (2021). Magnetically assisted drug delivery of topical eye drops maintains retinal function in vivo in mice. Pharmaceutics.

[91] Arvizo R, Bhattacharya R, Mukherjee P (2010). Gold nanoparticles: opportunities and challenges in nanomedicine. Expert Opin Drug Deliv.

[92] Maleki MJ, Ghasemi Y, Pourhassan-Moghaddam M, Asadi N, Dadashpour M, Abolghasem Mohammadi S (2019). Effect of green GO/Au nanocomposite on in-vitro amplification of human DNA. IET Nanobiotechnol.

[93] Cho W-K, Kang S, Choi H, Rho CR (2015). Topically administered gold nanoparticles inhibit experimental corneal neovascularization in mice. Cornea.

[94] Salem HF, Ahmed SM, Omar MM (2016). Liposomal flucytosine capped with gold nanoparticle formulations for improved ocular delivery. Drug Des Dev Ther.

[95] Hoshikawa A, Tagami T, Morimura C, Fukushige K, Ozeki T (2017). Ranibizumab biosimilar/polyethyleneglycol-conjugated gold nanoparticles as a novel drug delivery platform for age-related macular degeneration. J Drug Deliv Sci Technol.

[96] Maulvi FA, Patil RJ, Desai AR, Shukla MR, Vaidya RJ, Ranch KM (2019). Effect of gold nanoparticles on timolol uptake and its release kinetics from contact lenses: in vitro and in vivo evaluation. Acta Biomater.

[97] Dong Y, Wan G, Yan P, Qian C, Li F, Peng G (2019). Fabrication of resveratrol coated gold nanoparticles and investigation of their effect on diabetic retinopathy in streptozotocin induced diabetic rats. J Photochem Photobiol.

[98] Trigueros S, Domènech BE, Toulis V, Marfany G (2019). In vitro gene delivery in retinal pigment epithelium cells by plasmid DNA-wrapped gold nanoparticles. Genes.

[99] Ayata N, Sezer AD, Bucak S, Turanlı ET (2020). Preparation and in vitro characterization of monoclonal antibody ranibizumab conjugated magnetic nanoparticles for ocular drug delivery. Brazilian J Pharm Sci.

[100] Dave V, Sharma R, Gupta C, Sur S (2020). Folic acid modified gold nanoparticle for targeted delivery of Sorafenib tosylate towards the treatment of diabetic retinopathy. Colloids Surf B.

[101] Apaolaza P, Busch M, Asin-Prieto E, Peynshaert K, Rathod R, Remaut K (2020). Hyaluronic acid coating of gold nanoparticles for intraocular drug delivery: evaluation of the surface properties and effect on their distribution. Exp Eye Res.

[102] Sonntag T, Froemel F, Stamer WD, Ohlmann A, Fuchshofer R, Breunig M (2021). Distribution of gold nanoparticles in the anterior chamber of the eye after intracameral injection for glaucoma therapy. Pharmaceutics.

[103] Serati-Nouri H, Rasoulpoor S, Pourpirali R, Sadeghi-Soureh S, Esmaeilizadeh N, Dadashpour M (2021). In vitro expansion of human adipose-derived stem cells with delayed senescence through dual stage release of curcumin from mesoporous silica nanoparticles/electrospun nanofibers. Life Sci.

[104] Rosenholm MJ, Sahlgren C, Lindén M (2011). Multifunctional mesoporous silica nanoparticles for combined therapeutic, diagnostic and targeted action in cancer treatment. Curr Drug Targets.

[105] Wachter E, Dees C, Harkins J, Scott T, Petersen M, Rush RE (2003). Topical rose Bengal: Pre-clinical evaluation of pharmacokinetics and safety. Lasers Surg Med.

[106] Uppal A, Jain B, Gupta PK, Das K (2011). Photodynamic action of Rose Bengal silica nanoparticle complex on breast and oral cancer cell lines. Photochem Photobiol.

[107] Park J-H, Jeong H, Hong J, Chang M, Kim M, Chuck RS (2016). The effect of silica nanoparticles on human corneal epithelial cells. Sci Rep.

[108] Liao Y-T, Lee C-H, Chen S-T, Lai J-Y, Wu KCW (2017). Gelatin-functionalized mesoporous silica nanoparticles with sustained release properties for intracameral pharmacotherapy of glaucoma. J Mater Chem B.

[109] Kim S-N, Ko SA, Park CG, Lee SH, Huh BK, Park YH (2018). Amino-functionalized mesoporous silica particles for ocular delivery of brimonidine. Mol Pharm.

[110] Lin YX, Hu XF, Zhao Y, Gao YJ, Yang C, Qiao SL (2017). Photothermal ring integrated intraocular lens for high-efficient eye disease treatment. Adv Mater.

[111] Yang J, Gong X, Fang L, Fan Q, Cai L, Qiu X (2017). Potential of CeCl3@ mSiO2 nanoparticles in alleviating diabetic cataract development and progression. Nanomed Nanotechnol Biol Med.

[112] Hu C, Sun J, Zhang Y, Chen J, Lei Y, Sun X (2018). Local delivery and sustained-release of nitric oxide donor loaded in mesoporous silica particles for efficient treatment of primary open-angle glaucoma. Adv Healthc Mater.

[113] Nagai N, Yamaoka S, Fukuoka Y, Ishii M, Otake H, Kanai K (2018). Enhancement in corneal permeability of dissolved carteolol by its combination with magnesium hydroxide nanoparticles. Int J Mol Sci.

[114] Nagai N, Ogata F, Otake H, Kawasaki N, Nakazawa Y, Kanai K (2017). Co-instillation of nano-solid magnesium hydroxide enhances corneal permeability of dissolved timolol. Exp Eye Res.

[115] Peterson GI, Dobrynin AV, Becker ML (2017). Biodegradable shape memory polymers in medicine. Adv Healthc Mater.

[116] Di Colo G, Zambito Y, Zaino C, Sansò M (2009). Selected polysaccharides at comparison for their mucoadhesiveness and effect on precorneal residence of different drugs in the rabbit model. Drug Dev Ind Pharm.

[117] Lynch C, Kondiah PP, Choonara YE, du Toit LC, Ally N, Pillay V (2019). Advances in biodegradable nano-sized polymer-based ocular drug delivery. Polymers.

[118] Andrés-Guerrero V, Zong M, Ramsay E, Rojas B, Sarkhel S, Gallego B (2015). Novel biodegradable polyesteramide microspheres for controlled drug delivery in Ophthalmology. J Control Release.

[119] Aramwit P, Ekasit S, Yamdech R (2015). The development of non-toxic ionic-crosslinked chitosan-based microspheres as carriers for the controlled release of silk sericin. Biomed Microdevices.

[120] Mayol L, Biondi M, Russo L, Malle BM, Schwach-Abdellaoui K, Borzacchiello A (2014). Amphiphilic hyaluronic acid derivatives toward the design of micelles for the sustained delivery of hydrophobic drugs. Carbohydr Polym.

[121] Ahmed EM (2015). Hydrogel: preparation, characterization, and applications: a review. J Adv Res.

[122] Kirchhof S, Goepferich AM, Brandl FP (2015). Hydrogels in ophthalmic applications. Eur J Pharm Biopharm.

[123] Hernández R, Sacristán J, Asín L, Torres T, Ibarra M, Goya G (2010). Magnetic hydrogels derived from polysaccharides with improved specific power absorption: potential devices for remotely triggered drug delivery. J Phys Chem B.

[124] Balachandra A, Chan EC, Paul JP, Ng S, Chrysostomou V, Ngo S (2019). A biocompatible reverse thermoresponsive polymer for ocular drug delivery. Drug Deliv.

[125] Pandey V, Gajbhiye KR, Soni V (2015). Lactoferrin-appended solid lipid nanoparticles of paclitaxel for effective management of bronchogenic carcinoma. Drug Deliv.

[126] Rai A, Jain A, Jain A, Jain A, Pandey V, Chashoo G (2015). Targeted SLNs for management of HIV-1 associated dementia. Drug Dev Ind Pharm.

[127] Tekade RK, Maheshwari R, Tekade M, Chougule MB. Solid lipid nanoparticles for targeting and delivery of drugs and genes. In: Nanotechnology-Based Approaches for Targeting and Delivery of Drugs and Genes: Elsevier; 2017. p. 256-86. 10.1016/B978-0-12-809717-5.00010-5

[128] Balguri SP, Adelli GR, Majumdar S (2016). Topical ophthalmic lipid nanoparticle formulations (SLN, NLC) of indomethacin for delivery to the posterior segment ocular tissues. Eur J Pharm Biopharm.

[129] Amoabediny G, Haghiralsadat F, Naderinezhad S, Helder MN, Akhoundi Kharanaghi E, Mohammadnejad Arough J (2018). Overview of preparation methods of polymeric and lipid-based (niosome, solid lipid, liposome) nanoparticles: a comprehensive review. Int J Polym Mater.

[130] Mo Z, Ban J, Zhang Y, Du Y, Wen Y, Huang X (2018). Nanostructured lipid carriers-based thermosensitive eye drops for enhanced, sustained delivery of dexamethasone. Nanomedicine.

[131] Bhattacharjee A, Das PJ, Adhikari P, Marbaniang D, Pal P, Ray S (2019). Novel drug delivery systems for ocular therapy: with special reference to liposomal ocular delivery. Eur J Ophthalmol.

[132] Shi S, Peng F, Zheng Q, Zeng L, Chen H, Li X (2019). Micelle-solubilized axitinib for ocular administration in anti-neovascularization. Int J Pharm.

[133] Yadav M, Schiavone N, Guzman-Aranguez A, Giansanti F, Papucci L, de Lara MJP (2020). Atorvastatin-loaded solid lipid nanoparticles as eye drops: proposed treatment option for age-related macular degeneration (AMD). Drug Deliv Transl Res.

[134] Song K, Yan M, Li M, Geng Y, Wu X (2020). Preparation and in vitro–in vivo evaluation of novel ocular nanomicelle formulation of thymol based on glycyrrhizin. Colloids Surf B.

[135] Baig MS, Owida H, Njoroge W, Yang Y (2020). Development and evaluation of cationic nanostructured lipid carriers for ophthalmic drug delivery of besifloxacin. J Drug Deliv Sci Technol.

[136] Sood A, Gupta A, Agrawal G (2021). Recent advances in polysaccharides based biomaterials for drug delivery and tissue engineering applications. Carbohydr Polym technol Appl.

[137] Pathak K (2019). Marine bioadhesives: opportunities and challenges. Ther Deliv.

[138] Servais AB, Kienzle A, Valenzuela CD, Ysasi AB, Wagner WL, Tsuda A (2018). Structural heteropolysaccharide adhesion to the glycocalyx of visceral mesothelium. Tissue Eng Part A.

[139] George B, Suchithra T (2019). Plant-derived bioadhesives for wound dressing and drug delivery system. Fitoterapia.

[140] Irimia T, Ghica MV, Popa L, Anuţa V, Arsene A-L, Dinu-Pîrvu C-E (2018). Strategies for improving ocular drug bioavailability and corneal wound healing with chitosan-based delivery systems. Polymers.

[141] Nishikawa S, Tamai M (1996). Ultrastructure of hyaluronic acid and collagen in the human vitreous. Curr Eye Res.

[142] Nakagawa M, Tanaka M, Miyata T (1997). Evaluation of collagen gel and hyaluronic acid as vitreous substitutes. Ophthalmic Res.

[143] Fulgêncio GDO, Viana FAB, Ribeiro RR, Yoshida MI, Faraco AG, Cunha-Júnior ADS (2012). New mucoadhesive chitosan film for ophthalmic drug delivery of timolol maleate: in vivo evaluation. J Ocul Pharmacol Ther.

[144] Lodhi BA, Hussain MA, Ashraf MU, Farid-Ul-Haq M, Haseeb MT, Tabassum T (2020). Acute toxicity of a polysaccharide-based hydrogel from seeds of *Ocimum basilicum*. Cell Chem Technol.

[145] Dubashynskaya N, Poshina D, Raik S, Urtti A, Skorik YA (2020). Polysaccharides in ocular drug delivery. Pharmaceutics.

[146] Liu D, Lian Y, Fang Q, Liu L, Zhang J, Li J (2018). Hyaluronic-acid-modified lipid-polymer hybrid nanoparticles as an efficient ocular delivery platform for moxifloxacin hydrochloride. Int J Biol Macromol.

[147] Mittal N, Kaur G (2019). Investigations on polymeric nanoparticles for ocular delivery. Adv Polym Technol.

[148] Chaharband F, Daftarian N, Kanavi MR, Varshochian R, Hajiramezanali M, Norouzi P (2020). Trimethyl chitosan-hyaluronic acid nano-polyplexes for intravitreal VEGFR-2 siRNA delivery: formulation and in vivo efficacy evaluation. Nanotechnol Biol Med.

[149] Qian Q, Niu S, Williams GR, Wu J, Zhang X, Zhu L-M (2019). Peptide functionalized dual-responsive chitosan nanoparticles for controlled drug delivery to breast cancer cells. Colloids Surf A Physicochem Eng Asp.

[150] Lu T-Y, Huang W-C, Chen Y, Baskaran N, Yu J, Wei Y (2020). Effect of varied hair protein fractions on the gel properties of keratin/chitosan hydrogels for the use in tissue engineering. Colloids Surf B.

[151] Silva B, Marto J, São Braz B, Delgado E, Almeida AJ, Gonçalves L (2020). New nanoparticles for topical ocular delivery of erythropoietin. Int J Pharm.

[152] Yang D, So KF, Lo AC (2017). Lycium barbarum polysaccharide extracts preserve retinal function and attenuate inner retinal neuronal damage in a mouse model of transient retinal ischaemia. Clin Exp Ophthalmol.

[153] Chien KJ, Horng CT, Huang YS, Hsieh YH, Wang CJ, Yang JS (2018). Effects of Lycium barbarum (goji berry) on dry eye disease in rats. Mol Med Rep.

[154] Lakshmanan Y, Wong FSY, Zuo B, So K-F, Bui BV, Chan HHL (2019). Posttreatment intervention with lycium barbarum polysaccharides is neuroprotective in a rat model of chronic ocular hypertension. Invest Ophthalmol Vis Sci.

[155] Liu Y, Zhang Y (2019). Lycium barbarum polysaccharides alleviate hydrogen peroxide-induced injury by up-regulation of miR-4295 in human trabecular meshwork cells. Exp Mol Pathol.

[156] Liu L, Sha X-Y, Wu Y-N, Chen M-T, Zhong J-X (2020). Lycium barbarum polysaccharides protects retinal ganglion cells against oxidative stress injury. Neural Regen Res.

[157] Buosi FS, Alaimo A, Di Santo MC, Elías F, Liñares GG, Acebedo SL (2020). Resveratrol encapsulation in high molecular weight chitosan-based nanogels for applications in ocular treatments: impact on human ARPE-19 culture cells. Int J Biol Macromol.

[158] Luo L-J, Nguyen DD, Lai J-Y (2020). Dually functional hollow ceria nanoparticle platform for intraocular drug delivery: a push beyond the limits of static and dynamic ocular barriers toward glaucoma therapy. Biomaterials.

[159] Jiang P, Jacobs KM, Ohr MP, Swindle-Reilly KE (2020). Chitosan-polycaprolactone core–shell microparticles for sustained delivery of bevacizumab. Mol Pharm.

[160] Zoratto N, Forcina L, Matassa R, Mosca L, Familiari G, Musarò A (2021). Hyaluronan-cholesterol nanogels for the enhancement of the ocular delivery of therapeutics. Pharmaceutics.

[161] Wang S, Chi J, Jiang Z, Hu H, Yang C, Liu W (2021). A self-healing and injectable hydrogel based on water-soluble chitosan and hyaluronic acid for vitreous substitute. Carbohydr Polym.

[162] Kicková E, Sadeghi A, Puranen J, Tavakoli S, Sen M, Ranta V-P (2022). Pharmacokinetics of pullulan-dexamethasone conjugates in retinal drug delivery. Pharmaceutics.

[163] Sahle FF, Kim S, Niloy KK, Tahia F, Fili CV, Cooper E (2019). Nanotechnology in regenerative ophthalmology. Adv Drug Deliv Rev.

[164] Mitragotri S, Anderson DG, Chen X, Chow EK, Ho D, Kabanov AV (2015). Accelerating the translation of nanomaterials in biomedicine. ACS Nano.

[165] Tang J, Qin N, Chong Y, Diao Y, Wang Z, Xue T (2018). Nanowire arrays restore vision in blind mice. Nat Commun.

[166] Liu XL, Chen S, Zhang H, Zhou J, Fan HM, Liang XJ (2019). Magnetic nanomaterials for advanced regenerative medicine: the promise and challenges. Adv Mater.

[167] Hao R, Xing R, Xu Z, Hou Y, Gao S, Sun S (2010). Synthesis, functionalization, and biomedical applications of multifunctional magnetic nanoparticles. Adv Mater.

[168] Gao Y, Lim J, Teoh S-H, Xu C (2015). Emerging translational research on magnetic nanoparticles for regenerative medicine. Chem Soc Rev.

[169] Sharma R, Sharma D, Hazlett LD, Singh NK (2021). Nano-biomaterials for retinal regeneration. Nanomaterials.

[170] Karamichos D (2015). Ocular tissue engineering: current and future directions. J Funct Biomater.

[171] Masse F, Ouellette M, Lamoureux G, Boisselier E (2019). Gold nanoparticles in ophthalmology. Med Res Rev.

[172] Karakoçak BB, Raliya R, Davis JT, Chavalmane S, Wang W-N, Ravi N (2016). Biocompatibility of gold nanoparticles in retinal pigment epithelial cell line. Toxicol In Vitro.

[173] Leow S, Luu CD, Hairul Nizam M, Mok P, Ruhaslizan R, Wong H (2015). Safety and efficacy of human Wharton's Jelly-derived mesenchymal stem cells therapy for retinal degeneration. PLoS ONE.

[174] Yang J-W, Yu Z-Y, Cheng S-J, Chung JH, Liu X, Wu C-Y (2020). Graphene oxide–based nanomaterials: An insight into retinal prosthesis. Int J Mol Sci.

[175] Tummala GK, Joffre T, Lopes VR, Liszka A, Buznyk O, Ferraz N (2016). Hyperelastic nanocellulose-reinforced hydrogel of high water content for ophthalmic applications. ACS Biomater Sci Eng.

[176] Uzunalli G, Soran Z, Erkal TS, Dagdas YS, Dinc E, Hondur A (2014). Bioactive self-assembled peptide nanofibers for corneal stroma regeneration. Acta Biomater.

[177] Alarcon E, Vulesevic B, Argawal A, Ross A, Bejjani P, Podrebarac J (2016). Coloured cornea replacements with anti-infective properties: expanding the safe use of silver nanoparticles in regenerative medicine. Nanoscale.

[178] Kim JI, Kim JY, Park CH (2018). Fabrication of transparent hemispherical 3D nanofibrous scaffolds with radially aligned patterns via a novel electrospinning method. Sci Rep.

[179] Salehi S, Czugala M, Stafiej P, Fathi M, Bahners T, Gutmann JS (2017). Poly (glycerol sebacate)-poly (ε-caprolactone) blend nanofibrous scaffold as intrinsic bio-and immunocompatible system for corneal repair. Acta Biomater.

[180] Wu Z, Kong B, Liu R, Sun W, Mi S (2018). Engineering of corneal tissue through an aligned PVA/collagen composite nanofibrous electrospun scaffold. Nanomaterials.

[181] Nibourg LM, Gelens E, de Jong MR, Kuijer R, van Kooten TG, Koopmans SA (2016). Nanofiber-based hydrogels with extracellular matrix-based synthetic peptides for the prevention of capsular opacification. Exp Eye Res.

[182] Momenzadeh D, Baradaran-Rafii A, Keshel SH, Ebrahimi M, Biazar E (2017). Electrospun mat with eyelid fat-derived stem cells as a scaffold for ocular epithelial regeneration. Artif Cells Nanomed Biotechnol.

[183] Sharma R, Khristov V, Rising A, Jha BS, Dejene R, Hotaling N (2019). Clinical-grade stem cell–derived retinal pigment epithelium patch rescues retinal degeneration in rodents and pigs. Sci Transl Med.

[184] Thomas BB, Zhu D, Zhang L, Thomas PB, Hu Y, Nazari H (2016). Survival and functionality of hESC-derived retinal pigment epithelium cells cultured as a monolayer on polymer substrates transplanted in RCS rats. Investig Ophthalmol Vis Sci.

[185] Kashani AH, Uang J, Mert M, Rahhal F, Chan C, Avery RL (2020). Surgical method for implantation of a biosynthetic retinal pigment epithelium monolayer for geographic atrophy: experience from a phase 1/2a study. Ophthalmol Retina.

[186] Kashani AH, Lebkowski JS, Rahhal FM, Avery RL, Salehi-Had H, Dang W (2018). A bioengineered retinal pigment epithelial monolayer for advanced, dry age-related macular degeneration. Sci Transl Med.

[187] Fernández-Pérez J, Kador KE, Lynch AP, Ahearne M (2020). Characterization of extracellular matrix modified poly (ε-caprolactone) electrospun scaffolds with differing fiber orientations for corneal stroma regeneration. Mater Sci Eng C.

[188] Tayebi T, Baradaran-Rafii A, Hajifathali A, Rahimpour A, Zali H, Shaabani A (2021). Biofabrication of chitosan/chitosan nanoparticles/polycaprolactone transparent membrane for corneal endothelial tissue engineering. Sci Rep.

[189] Liu Y-C, Lin MTY, Ng AHC, Wong TT, Mehta JS (2020). Nanotechnology for the treatment of allergic conjunctival diseases. Pharmaceuticals.

[190] Zhao X, Si J, Huang D, Li K, Xin Y, Sui M (2020). Application of star poly (ethylene glycol) derivatives in drug delivery and controlled release. J Control Release.

[191] Srinivasarao DA, Lohiya G, Katti DS (2019). Fundamentals, challenges, and nanomedicine-based solutions for ocular diseases. Wiley Interdiscip Rev Nanomed Nanobiotechnol.

[192] Liu B, Kang C, Fang F (2020). Biometric measurement of anterior segment: a review. Sensors.

[193] Khiev D, Mohamed ZA, Vichare R, Paulson R, Bhatia S, Mohapatra S (2021). Emerging nano-formulations and nanomedicines applications for ocular drug delivery. Nanomaterials.

[194] Shen H-H, Chan EC, Lee JH, Bee Y-S, Lin T-W, Dusting GJ (2015). Nanocarriers for treatment of ocular neovascularization in the back of the eye: New vehicles for ophthalmic drug delivery. Nanomedicine.

[195] Besford QA, Cavalieri F, Caruso F (2020). Glycogen as a building block for advanced biological materials. Adv Mater.

[196] Nguyen DD, Lai J-Y (2020). Advancing the stimuli response of polymer-based drug delivery systems for ocular disease treatment. Polym Chem.

[197] Deshpande A, Mohamed M, Daftardar SB, Patel M, Boddu SH, Nesamony J. Solid lipid nanoparticles in drug delivery: Opportunities and challenges. In: Emerging nanotechnologies for diagnostics, drug delivery and medical devices, 2017, 291–330. 10.1016/B978-0-323-42978-8.00012-7

[198] Dhanasekaran S, Chopra S (2016). Getting a handle on smart drug delivery systems—a comprehensive view of therapeutic targeting strategies. Smart Drug Delivery System.

[199] Mohanta BC, Dinda SC, Palei NN, Deb J. Solid lipid based nano-particulate formulations in drug targeting. In: Role of novel drug delivery vehicles in nanobiomedicine, 2019, 95. 10.5772/intechopen.88268

[200] Poshina DN, Raik SV, Poshin AN, Skorik YA (2018). Accessibility of chitin and chitosan in enzymatic hydrolysis: a review. Polym Degrad Stab.

[201] Kritchenkov AS, Andranovitš S, Skorik YA (2017). Chitosan and its derivatives: vectors in gene therapy. Russ Chem Rev.

[202] Berezin A, Lomkova E, Skorik YA (2012). Chitosan conjugates with biologically active compounds: design strategies, properties, and targeted drug delivery. Russ Chem Bull.

[203] Tiwari S, Bahadur P (2019). Modified hyaluronic acid based materials for biomedical applications. Int J Biol Macromol.

[204] Fernando IS, Kim D, Nah J-W, Jeon Y-J (2019). Advances in functionalizing fucoidans and alginates (bio) polymers by structural modifications: a review. Chem Eng J.

[205] Pettignano A, Charlot A, Fleury E (2019). Carboxyl-functionalized derivatives of carboxymethyl cellulose: towards advanced biomedical applications. Polym Rev.

[206] Siafaka PI, Titopoulou A, Koukaras EN, Kostoglou M, Koutris E, Karavas E (2015). Chitosan derivatives as effective nanocarriers for ocular release of timolol drug. Int J Pharm.

[207] Zambito Y, Di Colo G (2010). Thiolated quaternary ammonium–chitosan conjugates for enhanced precorneal retention, transcorneal permeation and intraocular absorption of dexamethasone. Eur J Pharm Biopharm.

[208] Rassu G, Gavini E, Jonassen H, Zambito Y, Fogli S, Breschi MC (2009). New chitosan derivatives for the preparation of rokitamycin loaded microspheres designed for ocular or nasal administration. J Pharm Sci.

[209] Hume LR, Lee HK, Benedetti L, Sanzgiri YD, Topp EM, Stella VJ (1994). Ocular sustained delivery of prednisolone using hyaluronic acid benzyl ester films. Int J Pharm.

[210] Bongiovì F, Di Prima G, Palumbo FS, Licciardi M, Pitarresi G, Giammona G (2017). Hyaluronic acid-based micelles as ocular platform to modulate the loading, release, and corneal permeation of corticosteroids. Macromol Biosci.

[211] De Campos AM, Diebold Y, Carvalho EL, Sánchez A, José AM (2004). Chitosan nanoparticles as new ocular drug delivery systems: in vitro stability, in vivo fate, and cellular toxicity. Pharm Res.

[212] De Salamanca AE, Diebold Y, Calonge M, García-Vazquez C, Callejo S, Vila A (2006). Chitosan nanoparticles as a potential drug delivery system for the ocular surface: toxicity, uptake mechanism and in vivo tolerance. Invest Ophthalmol Vis Sci.

[213] Prow TW, Bhutto I, Kim SY, Grebe R, Merges C, McLeod DS (2008). Ocular nanoparticle toxicity and transfection of the retina and retinal pigment epithelium. Nanomed Nanotechnol Biol Med.

[214] Lai J-Y, Ma DHK, Cheng H-Y, Sun C-C, Huang S-J, Li Y-T (2010). Ocular biocompatibility of carbodiimide cross-linked hyaluronic acid hydrogels for cell sheet delivery carriers. J Biomater Sci Polym Ed.

[215] Zorzi GK, Párraga JE, Seijo B, Sánchez A (2011). Hybrid nanoparticle design based on cationized gelatin and the polyanions dextran sulfate and chondroitin sulfate for ocular gene therapy. Macromol Biosci.

[216] Lai J-Y (2012). Biocompatibility of genipin and glutaraldehyde cross-linked chitosan materials in the anterior chamber of the eye. Int J Mol Sci.

[217] Ogunjimi AT, Melo SM, Vargas-Rechia CG, Emery FS, Lopez RF (2017). Hydrophilic polymeric nanoparticles prepared from Delonix galactomannan with low cytotoxicity for ocular drug delivery. Carbohydr Polym.

[218] Etienne O, Schneider A, Taddei C, Richert L, Schaaf P, Voegel J-C (2005). Degradability of polysaccharides multilayer films in the oral environment: an in vitro and in vivo study. Biomacromol.

[219] Nguyen NTP, Nguyen LVH, Tran NMP, Nguyen DT, Nguyen TNT, Tran HA (2019). The effect of oxidation degree and volume ratio of components on properties and applications of in situ cross-linking hydrogels based on chitosan and hyaluronic acid. Mater Sci Eng C.

[220] Sultana S, Alzahrani N, Alzahrani R, Alshamrani W, Aloufi W, Ali A (2020). Stability issues and approaches to stabilised nanoparticles based drug delivery system. J Drug Target.

[221] Yu H, Wu W, Lin X, Feng Y (2020). Polysaccharide-based nanomaterials for ocular drug delivery: a perspective. Front Bioeng Biotechnol.

[222] Mehra NK, Cai D, Kuo L, Hein T, Palakurthi S (2016). Safety and toxicity of nanomaterials for ocular drug delivery applications. Nanotoxicology.

[223] Almeida H, Lobão P, Frigerio C, Fonseca J, Silva R, Sousa Lobo JM (2017). Preparation, characterization and biocompatibility studies of thermoresponsive eyedrops based on the combination of nanostructured lipid carriers (NLC) and the polymer Pluronic F-127 for controlled delivery of ibuprofen. Pharm Dev Technol.

[224] Zhang R, Qian J, Li X, Yuan Y (2017). Treatment of experimental autoimmune uveoretinitis with intravitreal injection of infliximab encapsulated in liposomes. Br J Ophthalmol.

[225] Tan G, Yu S, Pan H, Li J, Liu D, Yuan K (2017). Bioadhesive chitosan-loaded liposomes: a more efficient and higher permeable ocular delivery platform for timolol maleate. Int J Biol Macromol.

[226] Castro BFM, de Oliveira FG, Domingos LC, Cotta OAL, Silva-Cunha A, Fialho SL (2020). Positively charged polymeric nanoparticles improve ocular penetration of tacrolimus after topical administration. J Drug Deliv Sci Technol.

[227] Vaneev A, Tikhomirova V, Chesnokova N, Popova E, Beznos O, Kost O (2021). Nanotechnology for topical drug delivery to the anterior segment of the eye. Int J Mol Sci.

[228] Samimi M, Mahboobian M, Mohammadi M (2021). Ocular toxicity assessment of nanoemulsion in-situ gel formulation of fluconazole. Hum Exp Toxicol.

[229] Mehra N, Aqil M, Sultana Y (2021). A grafted copolymer-based nanomicelles for topical ocular delivery of everolimus: formulation, characterization, ex-vivo permeation, in-vitro ocular toxicity, and stability study. Eur J Pharm Sci.

[230] Bachu RD, Chowdhury P, Al-Saedi ZH, Karla PK, Boddu SH (2018). Ocular drug delivery barriers—role of nanocarriers in the treatment of anterior segment ocular diseases. Pharmaceutics.

[231] Eroglu YI (2017). A comparative review of Haute Autorité de Santé and National Institute for Health and Care Excellence health technology assessments of Ikervis® to treat severe keratitis in adult patients with dry eye disease which has not improved despite treatment with tear substitutes. J Mark Access Health Policy.

[232] Reimondez-Troitiño S, Csaba N, Alonso M, De La Fuente M (2015). Nanotherapies for the treatment of ocular diseases. Eur J Pharm Biopharm.

[233] Kalomiraki M, Thermos K, Chaniotakis NA (2016). Dendrimers as tunable vectors of drug delivery systems and biomedical and ocular applications. Int J Nanomedicine.

[234] Pooja D, Kadari A, Kulhari H, Sistla R. Lipid-based nanomedicines: Current clinical status and future perspectives. In: Lipid nanocarriers for drug targeting. Lipid-based nanomedicines. Elsevier; 2018. p. 509–28. 10.1016/B978-0-12-813687-4.00013-X.

[235] Palla S, Biswas J, Nagesha CK (2015). Efficacy of Ozurdex implant in treatment of noninfectious intermediate uveitis. Indian J Ophthalmol.

[236] Fusi-Rubiano W, Blow RR, Lane M, Morjaria R, Denniston AK (2018). Iluvien™(fluocinolone acetonide 0.19 mg intravitreal implant) in the treatment of diabetic macular edema: a review. Ophthalmol Ther.

[237] Kim HM, Woo SJ (2021). Ocular drug delivery to the retina: Current innovations and future perspectives. Pharmaceutics.

[238] Lee DJ (2015). Intraocular implants for the treatment of autoimmune uveitis. J Funct Biomater.

[239] Grumezescu AM (2018). Design of nanostructures for versatile therapeutic applications.

[240] Ghanchi F, Bourne R, Downes SM, Gale R, Rennie C, Tapply I (2022). An update on long-acting therapies in chronic sight-threatening eye diseases of the posterior segment: AMD, DMO, RVO, uveitis and glaucoma. Eye.

[241] Ma P, Mumper RJ (2013). Paclitaxel nano-delivery systems: a comprehensive review. J Nanomed Nanotechnol.

[242] Yang M, Peterson WM, Yu Y, Kays J, Cardona D, Culp D (2016). GB-102 for wet AMD: a novel injectable formulation that safely delivers active levels of sunitinib to the retina and RPE/choroid for over four months. Investig Ophthalmol Vis Sci.

[243] Gupta PK, Venkateswaran N (2021). The role of KPI-121 0.25% in the treatment of dry eye disease: penetrating the mucus barrier to treat periodic flares. Ther Adv Ophthalmol.

[244] Wong CW, Metselaar JM, Storm G, Wong TT (2021). A review of the clinical applications of drug delivery systems for the treatment of ocular anterior segment inflammation. Br J Ophthalmol.

[245] Bourlais C, Acar L, Zia HH, Sado PA, Needham T, Leverge R (1998). Prog Retin Eye Res.

[246] Gulsen D, Chauhan A (2004). Ophthalmic drug delivery through contact lenses. Investig Ophthalmol Vis Sci.

[247] Gaudana R, Jwala J, Boddu SH, Mitra AK (2009). Recent perspectives in ocular drug delivery. Pharm Res.

[248] Bochot A, Fattal E (2012). Liposomes for intravitreal drug delivery: a state of the art. J Control Release.

[249] Lee SJ, He W, Robinson SB, Robinson MR, Csaky KG, Kim H (2010). Evaluation of clearance mechanisms with transscleral drug delivery. Invest Ophthalmol Vis Sci.

[250] Patel A, Cholkar K, Agrahari V, Mitra AK (2013). Ocular drug delivery systems: An overview. World J Pharmacol.

[251] Zhang J, Jiao J, Niu M, Gao X, Zhang G, Yu H (2021). Ten years of knowledge of nano-carrier based drug delivery systems in ophthalmology: current evidence, challenges, and future prospective. Int J Nanomedicine.

[252] Nagarwal RC, Kant S, Singh P, Maiti P, Pandit J (2009). Polymeric nanoparticulate system: a potential approach for ocular drug delivery. J Control Release.

[253] Sharif NA (2021). Therapeutic drugs and devices for tackling ocular hypertension and glaucoma, and need for neuroprotection and cytoprotective therapies. Front pharmacol.

[254] Araújo J, Gonzalez E, Egea MA, Garcia ML, Souto EB (2009). Nanomedicines for ocular NSAIDs: safety on drug delivery. Nanomed Nanotechnol Biol Med.

[255] Amrite AC, Kompella UB (2005). Size-dependent disposition of nanoparticles and microparticles following subconjunctival administration. J Pharm Pharmacol.

[256] Cheruvu NP, Amrite AC, Kompella UB (2008). Effect of eye pigmentation on transscleral drug delivery. Invest Ophthalmol Vis Sci.

[257] Vadlapudi A, CholKAr K, Dasari S, Mitra A (2015). Ocular drug delivery. Drug Deliv.

[258] del Amo Páez EM. Ocular and systemic pharmacokinetic models for drug discovery and development. Academic Dissertation 2015. Hansaprint Printing House, Helsinki. ISBN 978-951-51-1425-9 (print)978-951-51-1426-6 (online).

[259] Schoenwald RD (1997). Ocular pharmacokinetics: Lippincott-Raven: Philadelphia.

[260] Mishima S, Gasset A, Klyce S, Baum J (1966). Determination of tear volume and tear flow. Invest Ophthalmol Vis Sci.

[261] Marsh DA, Kompella UB, Edelhauser HF (2011). Selection of drug delivery approaches for the back of the eye: opportunities and unmet needs. Drug product development for the back of the eye.

[262] Wilson CG, Tan LE, Mains J, Kompella UB, Edelhauser HF (2011). Principles of retinal drug delivery from within the vitreous. Drug product development for the back of the eye.

[263] Radhakrishnan K, Sonali N, Moreno M, Nirmal J, Fernandez AA, Venkatraman S (2017). Protein delivery to the back of the eye: barriers, carriers and stability of anti-VEGF proteins. Drug Discov Today.

[264] Kaji H, Nagai N, Nishizawa M, Abe T (2018). Drug delivery devices for retinal diseases. Adv Drug Deliv Rev.

[265] Agrahari V, Agrahari V, Mandal A, Pal D, Mitra AK (2017). How are we improving the delivery to back of the eye? Advances and challenges of novel therapeutic approaches. Expert Opin Drug Deliv.

[266] Lee SS, Hughes P, Ross AD, Robinson MR (2010). Biodegradable implants for sustained drug release in the eye. Pharm Res.

[267] Masadeh R, Obaidat R, Alsmadi MT, Altaani B, Khanfar M, Alshyab R (2018). Technical Insight into Biodegradable Polymers Used in Implants. Jordan J Pharm Sci.

[268] Tamboli V, Mishra GP, Mitra AK (2012). Biodegradable polymers for ocular drug delivery. Adv Ocul Drug Deliv.

[269] Kleiner LW, Wright JC, Wang Y (2014). Evolution of implantable and insertable drug delivery systems. J Control Release.

[270] García-Estrada P, García-Bon MA, López-Naranjo EJ, Basaldúa-Pérez DN, Santos A, Navarro-Partida J (2021). Polymeric implants for the treatment of intraocular eye diseases: trends in biodegradable and non-biodegradable materials. Pharmaceutics.

[271] Kompella UB, Edelhauser HF (2011). Drug product development for the back of the eye.

[272] Kanski JJ, Bowling B. Clinical ophthalmology: a systematic approach. Elsevier Saunders; 2011. 10.1016/B978-0-7020-4093-1.00019-7

[273] Varela-Fernández R, Díaz-Tomé V, Luaces-Rodríguez A, Conde-Penedo A, García-Otero X, Luzardo-Álvarez A (2020). Drug delivery to the posterior segment of the eye: Biopharmaceutic and pharmacokinetic considerations. Pharmaceutics.

[274] Smith S, Lorenz D, Peace J, McLeod K, Crockett R, Vogel R (2010). Difluprednate ophthalmic emulsion 0.05%(Durezol®) administered two times daily for managing ocular inflammation and pain following cataract surgery. Clin Ophthalmol.

[275] Park CH, Kim MK, Kim EC, Kim JY, Kim T-I, Kim HK (2019). Efficacy of topical cyclosporine nanoemulsion 0.05% compared with topical cyclosporine emulsion 0.05% and diquafosol 3% in dry eye. Korean J Ophthalmol.

[276] Leonardi A, Van Setten G, Amrane M, Ismail D, Garrigue J-S, Figueiredo FC (2016). Efficacy and safety of 0.1% cyclosporine A cationic emulsion in the treatment of severe dry eye disease: a multicenter randomized trial. J Ophthalmol.

[277] Mandal A, Gote V, Pal D, Ogundele A, Mitra AK (2019). Ocular pharmacokinetics of a topical ophthalmic nanomicellar solution of cyclosporine (Cequa®) for dry eye disease. Pharm Res.

[278] Buggage RR, Amrane M, Ismail D, Deniaud M, Lemp MA, Baudouin C (2012). The effect of cyclokat®(preservative-free cyclosporine 0.1% cationic emulsion) on dry eye disease signs and symptoms in sjogren and non-sjogren patients with moderate to severe DED in a phase III randomized clinical trial. Invest Ophthalmol Vis Sci.

[279] Бeздeткo П, Ильинa E (2017). Эффeктивнocть лeчeния пaтoлoгии пepeднeй пoвepxнocти глaзнoгo яблoкa пpeпapaтaми Эдeнopм 5% и Лaкpиceк oфтa плюc. Oфтaльмoлoгия Bocтoчнaя Eвpoпa.

[280] Garrigue J-S, Amrane M, Faure M-O, Holopainen JM, Tong L (2017). Relevance of lipid-based products in the management of dry eye disease. J Ocul Pharmacol Ther.

[281] Bressler NM, Bressler SB (2000). Photodynamic therapy with verteporfin (Visudyne): impact on ophthalmology and visual sciences. Investig Ophthalmol Vis Sci.

[282] Tobin KA (2006). Macugen treatment for wet age-related macular degeneration. Insight.

[283] Opitz DL, Harthan JS (2012). Review of azithromycin ophthalmic 1% solution (AzaSite®) for the treatment of ocular infections. Ophthalmol Eye Dis.

[284] Denis P, Baudouin C, Bron A, Nordmann J-P, Renard JP, Rouland JF (2010). First-line latanoprost therapy in ocular hypertension or open-angle glaucoma patients: a 3-month efficacy analysis stratified by initial intraocular pressure. BMC Ophthalmol.

[285] Benelli U (2011). Systane® lubricant eye drops in the management of ocular dryness. Clin Ophthalmol.

[286] Navratil T, Garcia A, Verhoeven RS, Trevino L, Gilger BC, Mansberger SL (2015). Advancing ENV515 (travoprost) intracameral implant into clinical development: nonclinical evaluation of ENV515 in support of first-time-in-human phase 2a clinical study. Invest Ophthalmol Vis Sci.

